# Comparisons of computational methods for differential alternative splicing detection using RNA-seq in plant systems

**DOI:** 10.1186/s12859-014-0364-4

**Published:** 2014-12-16

**Authors:** Ruolin Liu, Ann E Loraine, Julie A Dickerson

**Affiliations:** Department of Electrical and Computational Engineering, Iowa State University, Howe Hall, Ames, 50011-3060 USA; Department of Bioinformatics and Genomics, University of North Carolina at Charlotte, North Carolina Research Campus, 600 Laureate Way, Kannapolis, 28081 NC USA

**Keywords:** RNAseq, Alternative splicing, Plants

## Abstract

**Background:**

Alternative Splicing (AS) as a post-transcription regulation mechanism is an important application of RNA-seq studies in eukaryotes. A number of software and computational methods have been developed for detecting AS. Most of the methods, however, are designed and tested on animal data, such as human and mouse. Plants genes differ from those of animals in many ways, e.g., the average intron size and preferred AS types. These differences may require different computational approaches and raise questions about their effectiveness on plant data. The goal of this paper is to benchmark existing computational differential splicing (or transcription) detection methods so that biologists can choose the most suitable tools to accomplish their goals.

**Results:**

This study compares the eight popular public available software packages for differential splicing analysis using both simulated and real Arabidopsis thaliana RNA-seq data. All software are freely available. The study examines the effect of varying AS ratio, read depth, dispersion pattern, AS types, sample sizes and the influence of annotation. Using a real data, the study looks at the consistences between the packages and verifies a subset of the detected AS events using PCR studies.

**Conclusions:**

No single method performs the best in all situations. The accuracy of annotation has a major impact on which method should be chosen for AS analysis. DEXSeq performs well in the simulated data when the AS signal is relative strong and annotation is accurate. Cufflinks achieve a better tradeoff between precision and recall and turns out to be the best one when incomplete annotation is provided. Some methods perform inconsistently for different AS types. Complex AS events that combine several simple AS events impose problems for most methods, especially for MATS. MATS stands out in the analysis of real RNA-seq data when all the AS events being evaluated are simple AS events.

**Electronic supplementary material:**

The online version of this article (doi:10.1186/s12859-014-0364-4) contains supplementary material, which is available to authorized users.

## Background

Alternative splicing (AS) is a post-transcriptional regulation mechanism that allows a single gene to produce multiple mRNA transcripts. Some of the roles of AS include regulating gene expression in response to environmental stimuli and developmental changes [1-3]. In addition to contributing to protein diversity and regulation, some variants of AS may be nonfunctional and quickly degraded, providing gives cells another mechanism to regulate gene expression after transcription but before translation. AS occurs as a normal phenomenon in eukaryotes and is more abundant in higher eukaryotes than in lower eukaryotes [4]. More than 95% of human genes and 60% of Drosophila multi-exon genes are alternatively spliced [5]. In plants, 61% of intron-containing genes undergo alternative splicing [3].

Although there is no consensus classification of AS types, the five standard types are skipped exon (SE), alternative 3^′^ splice site (A3SS), alternative 5^′^ splice site (A5SS), mutually exclusive exons (MXE), and intron retention (IR) [6]. Animals and plants differ in their most common types of AS events. SE is the most common AS type in humans (>40*%*), but the least common type in plants (5%) [4]. Intron retention is the most prevalent AS type in plants (∼40*%*) but the least prevalent type in humans [7,8]. This difference suggests plants and animals may recognize exons and introns in different ways [7]. Also, AS does not always occur as one of the simple events described above; combinations of multiple simple AS events are common. In Arabidopsis, multiple exons may be skipped together and/or exon skipping occurs in the company of alternative 5’ and/or 3’ splice sites [8]. Such complex AS events are abundant in Arabidopsis latest annotation version, TAIR 10 [9].

Some evidence also suggests that plants and animals may regulate AS in different ways. For examples, plants possess nearly double the number of SR proteins as compared to nonphotosynthetic organisms [10]. SR stands for serine(S)-arginine(R)-rich proteins, a conserved family of pre-mRNA splicing factors. Interestingly, most SR proteins (14 of the 18 Arabidopsis SR protiens) [11] are themselves alternatively spliced and some studies have linked the AS of several SR proteins (e.g., SR45,SR45a,SR1/SR34, SR30) to environmental signals. AS is believed to play a critical role in helping plants adapt to their environment and may increase our understanding of plant and crop phenotypes [3].

The advent of RNA-seq has increased the observed frequency of AS in plants from 30% [12-14] in the pre-NGS era to 61% [8]. As RNA-seq becomes the new standard for studying gene and transcription expression, a key problem is to detect condition-specific differences, such as differential expression and differential alternative splicing. To date, dozens of methods for detecting differential AS using RNA-seq have been published. Most of the methods are designed for and tested on human, mouse and other mammals. Their performance on RNA-seq data from plants remains in question due to the differences in AS machinery between animals and plants. Recent review papers [15-17] compare differential alternative splicing detection methods with respect to methodology but do not evaluate performance under realistic conditions. Another two publications [18,19] benchmark methods and algorithms for transcript reconstruction and quantification. To our knowledge, this study is the first to systematically compare differential alternative splicing methods using RNA-seq in plant systems.

### Selection criteria and limitation of this study

This work benchmarks eight popular methods for differential AS according to the three criteria given below: effectiveness, biological replicates and software engineering.

- Effectiveness: the method should detect differential AS across samples. Note that this is not necessarily equivalent to isoform quantification problem as changes in the absolute isoform expression do not necessarily imply differential alternative splicing [15].
- Biological replicates: the selected method should be able to take advantage of biological replicates in the RNA-seq data sets.
- Software engineering: the method has to be implemented as a usable and robust program so that a scientist with limited computational skills can run the program regardless of understanding the theory behind it.

For example, under these criteria, some methods are ruled out for inclusion in this study. E.g., SpliceTrap [20] only quantifies alternative splicing within a single condition and MISO [21] and PSGInfer [22] do not support biological replicates. Our list of programs is not exhaustive; however, we have selected a set of programs which represent a variety of approaches. Due to our limited human resources and computational power, the current versions of FDM [23] and JuncBase [24] met our criteria but were excluded from this study. FDM uses a splice graph representation of aligned RNA-seq data and Jensen Shannon Divergence (JSD) to measure the difference in relative transcript abundances. JuncBase uses exclusively reads spanning exon-exon junctions. These concepts are well represented by the other methods we have compared in this study. Importantly, our testing pipeline and the input data needed to run the simulation are available in a Github repository, https://github.com/ruolin/ASmethodsBenchmarking. The whole pipeline is documented, interested readers can repeat the study and test the results with their preferred differential AS detection tools.

### Method classification

**Figure 1:**
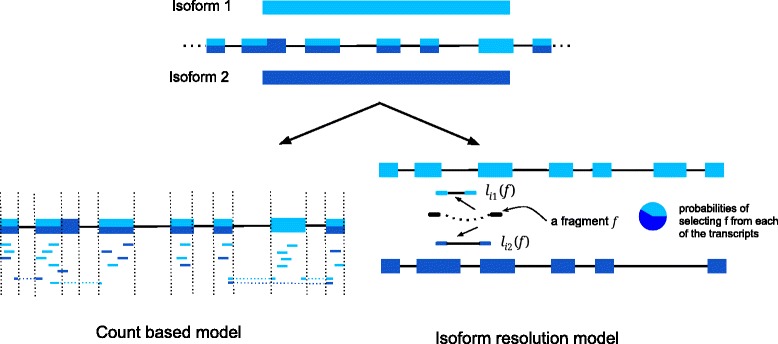
**Quantification schema.** A simplified gene model consists of two expressed isoforms (Top). Exons are colored according to the isoform of origin. Two model types used for quantification purpose (Bottom). In the count-based models (left), reads are assigned to counting units (shown by dash lines) without ambiguity. For each counting unit the model can be viewed as a test on two possible outcomes (spliced in or spliced out). The isoform resolution model is shown on the right where two ends of a read pair (show as dark solid boxes connected by curly dash line) align upstream and downstream of an alternative donor site. *l*_*i*1_(*f*) is the length of alignment of fragment *f* to isoform *i*1, and is shorter than *l*_*i*2_(*f*). Therefore if the fragment size distribution is known, it is possible to infer which isoform is more likely to generate *f*. Note that transcript effective length, i.e. *l*_*i*1_(*f*), *l*_*i*2_(*f*) and other parameters (depends on model you use) might also affect the probability of assigning reads to isoforms. Usually a maximum likelihood based approach is used to optimize this probability.

Methods for detecting AS may be categorized into two quantification schemas: count-based models and isoform resolution models (Figure 1). These two terms are based on the classification nomenclature defined by Pachter in [17]. We selected eight methods and evaluated them based on simulated and real data. Six of them are from count-based models: DEXSeq [25], DSGseq [26], SplicingCompass [27], MATS [28], rDiff-parametric [29] and SeqGSEA [30]. The remaining two, Cufflinks [31] and DiffSplice [32], use isoform resolution models. A brief overview of the eight methods follows.

### Count-based models

The count-based models are based on the methods used to quantify transcripts with single isoforms. The number of reads falling on a transcript (adjusted for transcript length and the total number of mapped reads), like RPKM (Reads Per Kilobase per Millions of reads mapped), is used as an estimate for abundance [17]. Count-based models are commonly used in differential gene expression. For differential splicing, the count-based models are modified to count reads in smaller counting units (i.e., exons) rather than the whole transcript regions. Also the focus changes to the differential expression of the counting units. Count-based models usually configure each gene into a single representation consisting of counting units. Counting units can be full or truncated exonic regions (e.g., DEXSeq and DSGseq), or junction regions (MATS). Although the count-based model does not directly address the issue of quantifying isoform abundances, the DSGseq authors prove that the reads at counting units can fully reflect isoform expression as long as there is no isoform that can be composed by the combination of other isoforms [26]. The count-based model can be seen as testing of two possible splicing outcomes, inclusion and/or exclusion, of each counting unit. Some papers refer to this model as an event-based model [15]. Methods using the count-based model are usually dependent on existing annotation on the gene structure and typically employ Poisson, generalized Poisson or Negative Binomial (NB) distributions to model the read counts on counting units. For RNA-seq, the NB distribution is considered better suited for the analysis of biological replicates than the Poisson distribution, as it is able to account for overdispersion in replicate counts [33,34].

SeqGSEA [30] and DSGseq [26] are examples of count-based models. These two methods are similar in many ways. Given a known set of transcripts at a locus, they both flatten these transcripts into a union transcript consisting of counting units (called mathematical exons in DSGseq and sub-exons in SeqGSEA). Both DSGseq and SeqGSEA model the number of reads that fall on the counting units as NB random variables after adjusting for overall gene expression. For a given gene, they calculate $\hat {p}_{\textit {ij}}$ as the expected read count fraction of counting units *i* in group *j* and variance of $\hat {p}_{\textit {ij}}$. Both methods define a gene-wise statistic to measure the difference in the expected read count fraction across two conditions by averaging over all counting units and adjusting for variance. Both methods mention that the null distribution is hard to obtain based on such statistics. SeqGSEA uses a permutation based approach to calculate the p-values while DSGseq just reports the statistics and does not calculate the p-values. Both DSGseq and SeqGSEA report which gene is alternatively spliced. A novel AS gene can be predicted only if an annotated constitutive exon is found to be a skipped exon. DSGseq can also tell you where the skipped exon may actually occur.

Like SeqGSEA and DSGseq, DEXSeq [25] transforms known gene models to sets of counting units (called counting bins in DEXSeq) based on any possible splice sites. The difference is that DEXSeq uses a generalized linear model (GLM) to detect the differential usage of counting units. The GLM in DEXSeq assumes a NB model for the counts. DEXseq reports which counting unit is alternatively used across conditions and, like SeqGSEA and DSGseq, a novel skipped exon can be predicted only on an annotated constitutive exon.

The rDiff [29] package consists of two methods: rDiff-parametric and rDiff-nonparametric. rDiff-parametric is a count-based model. Unlike other count-based methods it only makes inference on regions that are not shared among all isoforms (called alternative regions). rDiff-parametric uses the NB distribution to model the number of reads on counting units to account for biological variance. Unlike SeqGSEA and DSGseq, the variance is calculated from an empirical variance-mean relationship [29]. A p-value is calculated on each alternative region within a gene, and Bonferroni(BF) correction is used to obtain a genewise p-value. As a result, rDiff-parametric reports which gene is a significant AS gene but no novel AS gene can be found. The BF correction is known to be very stringent,which could explain why rDiff-parameteric has very low recall but high precision (see Results section).

MATS [28] first retrieves all AS events from input gene models and annotates the identified AS events with the corresponding AS types (e.g. SE, IR, A3SS). More specifically, it cannot detect novel AS events and only retrieves the simple AS events, not complex ones. MATS calculates a statistical metric called exon inclusion level, *ψ*, which is the proportion of the reads that exclusively support one outcome of the events to reads that exclusively support another outcome of the identified events. The exon inclusion level is always between 0 and 1. Then, the posterior probability of the difference of exon inclusion level across two samples which is larger than a user-defined cutoff, denoted *p*(|*ψ*_1_−*ψ*_2_|>*c* | *data*), is calculated. MATS reports which AS event is significant rather than which gene is alternatively spliced. MATS differs from other count-based model methods in that it uses Bayesian approaches. It is also the only method that does not assume independence of two biological conditions. A bivariate uniform prior is used to model the dependence. Information across genes is borrowed in the process of estimating the common prior. Although the method in MATS’s original paper is only designed for a two sample comparison, the latest version of MATS (3.0+) accepts multiple replicates. However, it is unclear how the program models biological variability.

Like DEXSeq and DSGseq, SplicingCompass [27] uses a union transcript model for each gene. However, it does not utilize any statistical model based on the counting process. SplicingCompass first constructs vectors of read counts on exons as well as on splicing junctions for each gene and sample, then calculates pairwise geometric angles between two vectors. Finally, a one-sided t-test comparing the within condition angles and between condition angles is carried out for each gene. SplicingCompass reports which gene is AS gene based on the t-test. Therefore a novel AS gene can be found if the aforementioned test turns out to be significant. Again only SE can be detected.

### Isoform resolution models

Isoform resolution models (also called multi-read models [17]) are multi-isoform models. Instead of transforming the question into detecting differential usage of counting units, they seek to directly solve this problem by comparing the relative isoform abundance across samples and/or conditions. The estimation of the isoform proportion vector *q* is usually done by maximizing a likelihood function *L*(*q*|*observingasetofreadsalignments*). Maximizing this likelihood function is equivalent to maximizing the likelihood of selecting a read or fragment from a transcript [31]. Isoform resolution models try to assign reads or fragments to the transcripts they came from at the cost of introducing additional uncertainty in read assignments due to the overlap between isoforms. In count-based models there is no ambiguity in assigning reads toward counting units. It is worth mentioning that this question is also connected to the question of transcriptome assembly as novel transcripts are found in nearly every RNA-seq study [17].

Cufflinks [31] and DiffSplice [32] are examples of the isoform resolution models. Cufflinks contains three independent but connected programs: Cufflinks, Cuffmerge and Cuffdiff. Cufflinks assembles and quantifies the aligned reads while Cuffdiff performs differential testing. Cufflinks uses a linear model [31] which includes a specific parameter for fragment length. This differentiates Cufflinks from other methods by allowing Cufflinks to take advantage of insert size information in paired-end data. In this sense, Cufflinks is more appropriate for paired-end reads. The estimate of relative abundance of a transcript is reported in the form of FPKM (fragments per kilobase per million mapped fragments) which is equivalent to RPKM in the single-end case. Cuffdiff performs tests for relative isoform abundance changes (called post-transcriptional overloading in the Cufflinks paper) using a one-sided t-test of the Jensen-Shannon Divergence metric [31]. Cufflinks is able to assemble transcriptomes and is thus less dependent on the accuracy of gene annotation.

Rigorously speaking, DiffSplice[32] is not “Isoform resolution” but “alternative paths resolution”. In DiffSplice, the alternative paths stand for the paths from the Alternative Spliced Module (ASM) in spliced graphs and each ASM has at least two alternative paths. An ASM is a region in splice graphs where isoforms differ from each other. ASM seeks to minimize the ambiguity in isoform resolution by only considering regions that are not shared by all isoforms. DiffSplice tests differential splicing on each ASM instead of whole transcripts. The relative abundances of alternative paths are estimated using the maximum likelihood method. The difference of the relative abundances compositions is measured using Jensen-Shannon Divergence metric (JSD). Both the DiffSplice and Cufflinks models are extensions of the model of [35]. Cufflinks extends the model to the paired-end case while DiffSplice restricts it to ASMs. Like Cufflinks, DiffSplice is also capable of assembling the aligned reads onto the transcriptome. Therefore, both programs are able to detect novel AS events that are not in the annotation. However, the Cuffmerge from Cufflinks packages can merge the assembly with annotations to provide gene models with higher confidence while no previous knowledge of gene models is used by DiffSplice. In other words, annotation is not used in DiffSplice.

## Results and discussion

These differential AS detection methods were first evaluated using simulated data with known ground truth, where we could control the level of differential splicing across conditions and other factors that may affect detection. The NB distributions were used to simulate read counts on genes. The mean and dispersion parameters for the NB distributions were estimated from heat shock data [36]. The 5885 genes that are known to have at least two splice variants in the Arabidopsis TAIR 10 reference annotation were focused on in the simulation studies. Using our custom simulation pipeline (see Additional file 1), a set of 2000 genes was randomly chosen from the overlaps between the 5885 known AS genes and genes that have non-zero expression in real data sets. These 2000 genes were simulated to be alternatively spliced and are referred to as “true AS genes”. Details about the simulation settings and procedures can be found in the Methods section.

In the simulation study, we evaluated the robustness of the methods by varying the degree of differential splicing, read depths, sample sizes and dispersion setting in different conditions. We set High, Medium and Low levels for AS ratio, two dispersion patterns and three levels of read depth (100×,60× and 25×). In addition, we have compared the computational time required for running the analysis (Additional file 1: Table S1). We used two dispersion settings in the simulation. One allows the two conditions to use two different dispersion parameters in the NB distributions which are estimated from two replicated real RNA-seq data sets, whereas the other forces both conditions to have the same dispersion parameter which is estimated from the pooled RNA-seq data sets. We call these two settings different dispersion pattern versus same dispersion pattern (denoted by Diff vs Same). We also investigated the effect of sample size, from 3 to 8 samples per conditions. A simple notation $\textrm {\small {High}}^{\text {Diff}}_{\textrm {100}\times }$ means a condition of read depth at 100, different dispersion pattern and high AS ratio across conditions.

**Figure 2:**
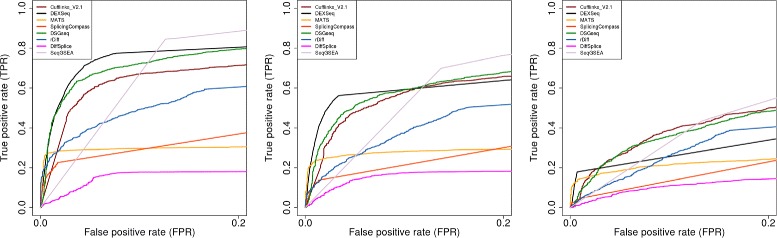
**ROC curves evaluation for three levels of AS ratio when two groups of samples have the different dispersion pattern.** ROC curves for eight selected methods in simulation studies $\textrm {\small {High}}^{\text {Diff}}_{\textrm {100x}}$ (left panel), $\textrm {\small {Medium}}^{\text {Diff}}_{\textrm {100x}}$ (middle panel), $\textrm {\small {Low}}^{\text {Diff}}_{\textrm {100x}}$ (right panel). These ROC curves are obtained at a simple size of 3 for each condition. When the level or degree of DS across conditions become smaller (panel left-right), the power of discrimination of true-DS and non-DS drops significantly. However the relative ranking of each methods tend to be unchanged. DEXSeq perform consistently the best with respect to all three simulation studies.

**Figure 3:**
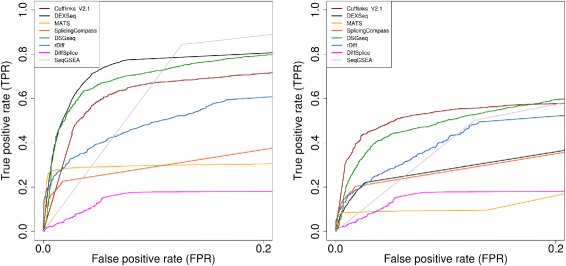
**ROC curves evaluation for accurate and incomplete annotation.** ROC curves for eight selected methods using simulation study $\textrm {\small {High}}^{\text {Diff}}_{\textrm {100x}}$ with complete annotation (left panel) and incomplete annotation (right panel). Isoform resolution model methods, such as Cufflinks, are more robust to incomplete annotation compared with count-based models methods.

**Figure 4:**
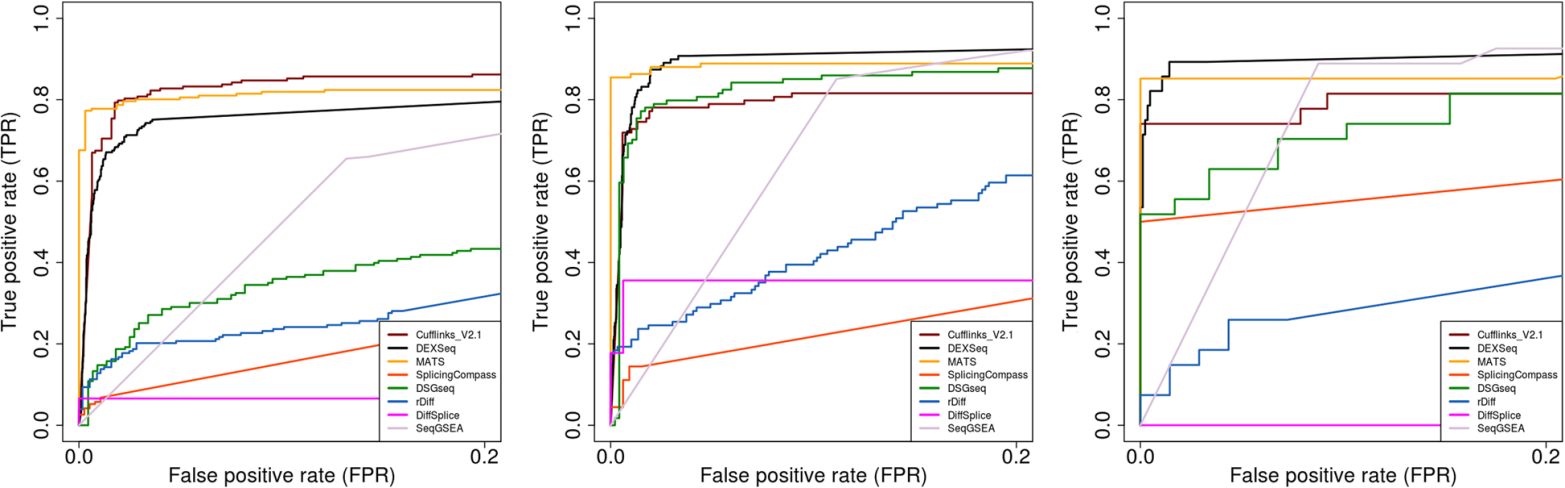
**ROC curves evaluations for three splicing classes.** ROC curves of eight selected methods based on 1755 genes containing single splicing event from simulation study $\textrm {\small {High}}^{\text {Diff}}_{\textrm {100x}}$. These 1755 genes were further divided into three splicing event classes: 803 genes with alt. donor/acceptor sites (left panel), 850 genes with intron retention (middle panel), 102 genes with exon skipping (right panel).

All of these evaluations were carried out in terms of the Receiver Operating Characteristic (ROC) curves and the Area Under the Curve (AUC) metric. The ROC curve depicts the true-positive rate (TPR) of a method for different false-positive rates (FPR) by varying the threshold for given scores. TPR is defined as the proportion of the events that are known to be differentially spliced that test as positives. Similarly the FPR is the proportion of the events that are known not to have differential splicing that test as positives. As almost all AS detection software packages tightly control FPR, we restricted the ROC curves to the range of 0−0.2 (Figures 2, 3 and 4). The area under the ROC curve, or AUC, is the numerical measurement that summarizes the ROC curves. Here we calculated the AUC under the restricted ROC curves. Methods with larger AUC have better performance. The results of all simulation studies under the measurement of AUC are summarized in Table 1.

**Table 1:** **Area under the ROC curve (AUC) and relative ranking measured under all simulation studies**

	**Cufflinks**	**DEXSeq**	**MATS**	**SpComp**	**DSGseq**	**rDiff-param**	**DiffSplice**	**SeqGSEA**
$\textrm {\small {High}}^{\text {Diff}}_{\textrm {100x}}$	0.7765(3)	**0.8435(1)**	0.6066(7)	0.603(6)	0.8214(2)	0.704(5)	0.5262(8)	0.7699(4)
$\textrm {\small {Medium}}^{\text {Diff}}_{\textrm {100x}}$	0.7334(3)	**0.7583(1)**	0.5960(6)	0.5612(7)	0.7472(2)	0.6421(5)	0.5276(8)	0.7055(4)
$\textrm {\small {Low}}^{\text {Diff}}_{\textrm {100x}}$	**0.6369(1)**	0.5847(4)	0.5583(6)	0.518(7)	0.6288(2)	0.5807(5)	0.4982(8)	0.6155(3)
$\textrm {\small {High}}^{\text {Same}}_{\textrm {100x}}$	0.7751(4)	0.8351(2)	0.6046(6)	0.5998(7)	**0.8373(1)**	0.6871(5)	0.5371(8)	0.7797(3)
$\textrm {\small {Medium}}^{\text {Same}}_{\textrm {100x}}$	0.7357(4)	0.7407(2)	0.5914(6)	0.5582(7)	**0.7669(1)**	0.6201(5)	0.5341(8)	0.7374(3)
$\textrm {\small {Low}}^{\text {Same}}_{\textrm {100x}}$	0.6487(2)	0.5546(5)	0.5506(6)	0.5159(7)	**0.6496(1)**	0.5773(4)	0.5049(8)	0.6297(3)
$\textrm {\small {100x}}^{\text {Diff}}_{\text {High}}$	0.7765(3)	**0.8435(1)**	0.6066(7)	0.603(6)	0.8214(2)	0.704(5)	0.5262(8)	0.7699(4)
$\textrm {\small {\ 60x}}^{\text {Diff}}_{\text {High}}$	**0.8687(1)**	0.7667(2)	0.5861(6)	0.5688(7)	0.7648(3)	0.6848(5)	0.5266(8)	0.7338(4)
$\textrm {\small {\ 25x}}^{\text {Diff}}_{\text {High}}$	0.6807(4)	**0.7432(1)**	0.5607(6)	0.5479(7)	0.6967(2)	0.6659(5)	0.5001(8)	0.6815(3)
Complete annot.	0.7765(3)	**0.8435(1)**	0.6066(7)	0.603(6)	0.8214(2)	0.704(5)	0.5262 (8)	0.7699 (4)
Incomplete annot.	**0.7271(1)**	0.5939(5)	0.5012(8)	0.5930(6)	0.7033(2)	0.6561(3)	0.5262 (7)	0.6425 (4)
A3A5SS	**0.8990(1)**	0.8574(3)	0.8948(2)	0.5283(7)	0.6272(5)	0.5732(6)	0.4811(8)	0.6932(4)
IR	0.8810(4)	**0.9368(1)**	0.9360(2)	0.5639(8)	0.8990(3)	0.6696(6)	0.6391(7)	0.7940(5)
SE	0.8795(3)	**0.9407(1)**	0.9177(2)	0.7500(6)	0.8301(5)	0.5916(7)	0(8)	0.8334(4)
8samples	0.7408(5)	**0.8495(1)**	0.6078(7)	0.7450(4)	0.8301(2)	0.7196(6)	0.5030(8)	0.7656(3)

Larger values of AUC indicate better performance. The table contains the AUC and relative ranking for the methods under all simulation study. The best method under each study is highlighted in boldface. The ranking position is shown in the parenthesis. A3A5SS stands for the joint class of alternative 3’ splice site event and alternative 5’ splice site event. IR stands for intron retention event and SE stands for skipping exon event.

As ROC curves and AUC measure the discrimination power between non-differentially spliced (non-DS) gene and differentially spliced (DS) gene over an interval, scientists are often interested in the discrimination power at a single cutoff point. Therefore the recall and precision at a *P*_*adj*_=0.05 cutoff were used as a additional set of evaluation metrics (*P*_*adj*_ stands for multiple testing corrected p-value). Recall is equivalent to TPR while precision is the proportion of the events that test as positives that are actually true discoveries. Precision is also known as 1−*falsediscoveryrate*. Evaluating on precision examines whether the methods are able to control the FDR at the claimed 0.05 level. DSGseq does not return p-values and was excluded from this evaluation and SeqGSEA did not report any gene under *FDR*=0.05 when the sample size was 3. The results of other seven methods under the measurement of recall and precision or FDR at *P*_*adj*_=0.05 are summarized in Table 2.

**Table 2:** **Recall and precision at** ***P***_***adj***_ ***=0.05*** **measured under all simulation studies**

	**Cufflinks**		**DEXSeq**		**MATS**		**SpComp**		**rDiff-param**		**DiffSplice**		**SeqGSEA**	
	**Rec.**	**Prec.**	**Rec.**	**Prec.**	**Rec.**	**Prec.**	**Rec.**	**Prec.**	**Rec.**	**Prec.**	**Rec.**	**Prec.**	**Rec.**	**Prec.**
$\textrm {\small {High}}^{\text {Diff}}_{\textrm {100x}}$	0.57	0.91	0.53	0.65	0.28	0.98	0.14	0.95	0.06	0.99	0.24	0.79	-	-
$\textrm {\small {Medium}}^{\text {Diff}}_{\textrm {100x}}$	0.40	0.91	0.31	0.71	0.22	0.98	0.08	0.90	0.02	0.95	0.24	0.76	-	-
$\textrm {\small {Low}}^{\text {Diff}}_{\textrm {100x}}$	0.03	0.77	0.06	0.59	0.1	0.99	0.02	0.82	0.002	0.833	0.20	0.66	-	-
$\textrm {\small {High}}^{\text {Same}}_{\textrm {100x}}$	0.58	0.90	0.49	0.71	0.27	0.98	0.13	0.94	0.05	1.0	0.26	0.84	-	-
$\textrm {\small {Medium}}^{\text {Same}}_{\textrm {100x}}$	0.42	0.91	0.25	0.80	0.21	0.99	0.07	0.92	0.01	1.0	0.25	0.81	-	-
$\textrm {\small {Low}}^{\text {Same}}_{\textrm {100x}}$	0.15	0.91	0.04	0.96	0.08	0.99	0.02	0.84	0.001	1.0	0.21	0.68	-	-
$\textrm {\small {100x}}^{\text {Diff}}_{\text {High}}$	0.57	0.91	0.53	0.65	0.28	0.98	0.14	0.95	0.06	0.99	0.24	0.79	-	-
$\textrm {\small {\ 60x}}^{\text {Diff}}_{\text {High}}$	0.49	0.91	0.29	0.72	0.22	0.99	0.09	0.93	0.02	1.0	0.25	0.81	-	-
$\textrm {\small {\ 25x}}^{\text {Diff}}_{\text {High}}$	0.39	0.92	0.22	0.75	0.15	0.98	0.06	0.93	0.008	0.94	0.17	0.79	-	-
A3A5SS	0.73	0.95	0.71	0.71	0.85	1	0.04	0.875	0.01	1	0.07	0.85	-	-
IR	0.69	0.95	0.43	0.8	0.76	0.99	0.09	0.8	0.09	1	0.36	0.82	-	-
SE	0.67	1	0.71	0.91	0.85	1	0.38	1	0.04	1	0	0	-	-
Complete annot.	0.57	0.91	0.53	0.65	0.28	0.98	0.14	0.95	0.06	0.99	0.24	0.79	-	-
Incomplete annot.	0.67	0.92	0.14	0.41	0.08	0.97	0.12	0.93	0.008	0.94	0.24	0.79	-	-
8samples	0.65	0.81	0.66	0.55	0.3	0.93	0.50	0.82	0.06	0.99	0.17	0.72	0.95	0.58

A3A5SS stands for the joint class of alternative 3’ splice site event and alternative 5’ splice site event. IR stands for intron retention event and SE stands for skipping exon event. Recalls were shown as the numbers in the left column, precisions in the right column. Larger values of both metrics are better. Under a sample size of 3, SeqGSEA found no genes at ***P***_***adj***_ ***=0.05*** and therefore no values were reported.

For the real data, we first compared the results obtained by the different methods in terms of absolute number of significant AS gene calling, the overlap of results across software and the concordance of gene rankings. We further compared these results to a list of experimentally validated genes that are known to be alternatively spliced in response to ambient temperature changes. Finally we carried out an semi-RT-PCR study and compared the results of the computational methods using RNA-seq to the results from RT-PCR.

### The effect of different levels of AS ratio in conjunction with dispersion pattern

Since the difference required between two isoform compositions to be biologically significant enough to call as differential splicing is an open question, we defined a parameter *PALT* (Percentage of ALTernative isoform) to control the level of differential splicing in our simulation. *PALT*, whose range is from 0−1, simply represents the relative abundances of alternative isoforms for given genes. For multi-transcript genes, we randomly chose one transcript as an alternative isoform while the rest of isoforms remained as standard isoforms across conditions. For each of given genes, all standard isoforms have relative abundances which summed to 1−*PALT*. The PALT for 2000 true AS genes was set to 0.2 in the control group and 0.4, 0.6, 0.8 in the three treatment groups, corresponding to low, medium and high AS ratio levels. We investigated the effect of varying the AS ratio level under two dispersion patterns. As a result we carried out 6 simulation studies and denoted them in the format of $\textrm {\small {High}}^{\text {Diff}}_{\textrm {100x}}$, representing the situations for high AS ratio, different dispersion patterns for two conditions and 100x read depth.

The restricted ROC curves of the 8 selected methods based on 3 simulation studies on different dispersion patterns are shown in Figure 2. As *PALT* changed from 0.8 to 0.4, the difference between the isoform compositions under the two simulated conditions became smaller. All methods lost their discrimination power as the signal of differential splicing became weaker. The results from simulation studies with the same dispersion pattern were similar and are shown in the (Additional file 1: Figure S9). When two simulated conditions had different dispersion patterns, DEXSeq performed well in high and medium AS ratio situations but not in the low AS ratio situation. (Figure 2 and Table 1). When two conditions had the same dispersion pattern, DSGseq consistently performed the best out of the 8 methods (Table 1). As we focused on the low AS ratio in both dispersion situations, Cufflinks performed the best.

Both AUC and recalls were affected by the change of the AS ratio but the effect on recalls seemed to be larger. Taking Cufflinks as an example, the recall rates were 57%, 40% and 3% at high, medium and low levels of differential splicing respectively (Table 2). However the AUC dropped only 14% from high to low alternative splicing ratio (Table 1). It is not surprising that AUC is a more robust measurement than recall and precision. But it is not uncommon for people to use a single cutoff point, e.g. declare significance at *FDR*=0.05. In this sense, the low AS ratio has a severe impact on the discrimination power (Table 2). DiffSplice achieved the highest recall in both $\textrm {\small {Low}}^{\text {Diff}}_{\textrm {100x}}$ and $\textrm {\small {Low}}^{\text {Same}}_{\textrm {100x}}$. However, its performance under the measurement of AUC (Table 1) was far from satisfactory since many AS events were not detected by using ASM and some detected ASMs were simply artifacts. In the baseline simulation study $\textrm {\small {High}}^{\text {Diff}}_{\textrm {100x}}$, 2123 ASMs were reported by DiffSplice and 94 of them resided at least 1kb away from coding regions. 4 ASMs were even longer than the longest gene (which is 31257 nt long) in Arabidopsis TAIR 10 model.

When considering the ability to control for false discoveries, all methods except MATS performed more poorly when the AS ratio became smaller (Table 2). Only MATS was able to control the FDR at all levels of AS ratio and dispersion pattern. SplicingCompass and rDiff-parametric could control the FDR at the desired 0.05 level in the simulation studies with high AS ratio but failed at low AS ratio, low levels of coverage. DEXSeq and rDiff-parametric’s abilities to control FDR improved if the data shared the same dispersion pattern across conditions. With same dispersion pattern, rDiff-parametric was able to perfect control the FDR in all three AS ratios while DEXSeq achieved the desired FDR level on low AS ratio but not on high AS ratio. Although DEXSeq had the best performance in terms of AUC, it did a poor job in controlling the FDR (Table 2).

### Detecting novel splicing events

We simulated RNA-seq reads using the latest Arabidopsis TAIR 10 gene sequences and models. This implies that no AS event is novel to this annotation. Theoretically methods that use annotation information should be able to find all candidate AS regions provided the annotation is correct. However in a real RNA-seq study, even in model organisms, there may be many novel splicing events. To simulate this case, we deliberately removed the mRNA model of the alternative transcripts from annotation for the set of true AS genes. The relative abundances of alternative transcripts are controlled by *PALT* and are the dominant force in the simulated AS events. By running the software using this incomplete annotation, we evaluated their abilities to detect novel splicing events. This comparison was evaluated on the baseline simulation study $\textrm {\small {High}}^{\text {Diff}}_{\textrm {100x}}$ (Figure 3). Except for DiffSplice, the performances of all other methods were degraded. Because DiffSplice does not use annotation information, its performance did not change. Overall, Cufflinks was more robust to incomplete annotation than other methods. MATS and DEXSeq’s performances dropped significantly, suggesting that these two methods are very dependent on accurate annotation.

### The effect of different AS types

Based on the gene models and sequences of the 5885 annotated AS genes in TAIR 10 annotation, we simulated 2000 true AS genes to be differentially spliced. However, most of the genes (1335 out of 2000, 67%) have more than one AS type. This made testing the performance in terms of the effect of different AS types difficult. Also as some methods, e.g. MATS and DiffSplice, test on individual events or local regions while others work on the gene level, the previous comparisons were not based on common ground. To overcome these problems, we picked out 1755 genes that have exactly two transcripts and a single splicing event from the 5885 genes. We then reevaluated all methods on these 1755 genes in the baseline simulation study. This equated the detection on a gene level to the detection on a splicing event. We classified these 1755 genes into three new sets by their splicing event types which include exon skipping, intron retention and alternative donor/acceptor sites (Figure 4). We treated alternative donor sites and acceptor sites together as a single class because there is almost no difference in detecting them from mathematical and computational perspective. 803 genes had an alternative donor or acceptor event, 850 showed intron retention and 102 demonstrated exon skipping and about one third of genes in each new set were pre-selected AS genes (274, 275 and 38 respectively). We evaluated the eight methods in each category. This is a simplified scenario where a gene has exactly one AS event.

DEXSeq achieved the highest AUC in two of the three simple event classes, IR and SE, (Table 1). In these two cases, the exons or introns are either included or excluded as a whole. However in the cases of A3SS and A533, the counting units could be as short as several bps. DEXSeq may not have enough read counts to perform reliable statistical tests in such short regions. We observed that Cufflinks which uses isoform-resolution models perform the best for A3SS and A5SS. When the complex AS events were excluded MATS’s improvement was very significant. The averaged AUC for MATS was 0.5763 when complex AS events were included. While it averaged at 0.9143 in the simplified scenarios (Table 1). This agrees with our observation that MATS is not capable of discovering complex AS events. In the simple scenario MATS acquired the highest recall and lowest FDR at *P*_*adj*_=0.05 threshold in all simple AS events (Table 2). As we looked at the individual types of AS events, DSGseq performed well for detecting IR but not so well on other splicing types. Similarly, Cufflinks performed well at A3SS and A5SS but poorly with other AS types, indicating a bias in detecting different AS types.

### The effect of sample sizes and read depth

The increase in sample size from 3 to 8 did not have a significant impact on the AUC statistics and the methods’ rankings based on the AUC (Table 1). Even for the recall and precision statistics (Table 2), the increase in sample size had a small impact for all methods except for SplicingCompass and SeqGSEA. Recall for SplicingCompass increased from 14% to 50% when the sample size increased from 3 to 8. SeqGSEA was not statistically significant at *FDR*=0.05 for a sample size of 3 but achieved a recall of 95% at the cost of having a low precision (58%) in a sample size of 8. However the ROC curves and AUC statistics for SeqGSEA were almost the same for the different sample sizes (Additional file 1: Figure S10). A possible explanation is that the permutation-based approach used in SeqGSEA may scale the *P*_*adj*_ according to the sample size. Therefore, we would recommend a sample size between 4 to 7 for using SeqGSEA.

Most methods were robust to different read depths or coverage of RNA-seq with a minor drop of discrimination power as read depth decreased (Table 1 and Additional file 1: Figure S11). However it is interesting to note that Cufflinks achieves its best discrimination power at *RD60* and ranked 1st among all methods at this read depth (Table 1). This may suggest that Cufflinks performs better when read depth is around 60.

### Real RNA-seq data from Arabidopsis heat shock experiment

In addition to the simulated data, we also evaluated the methods on heat shock RNA-seq data sets [36]. The results of eight selected programs on real data are given in the Additional file 2. Three RNA-seq samples were generated from heat shock T1 group and two from control T1 group (See Methods for a description for the heat shock data sets). All the eight methods except for DiffSplice are able to handle the unbalanced design with different sample sizes. For DiffSplice, we took out one sample from the heat stress group to make it a balanced design. All genes found to be AS at the threshold of *FDR*=0.05 were consider statistically significant. DSGseq does not report a p-value and therefore was not used for this comparison.

We first compared the number of significant AS events found by each method (Table 3). SeqGSEA did not find any gene with significant AS. This result was consistent with our simulation studies that SeqGSEA usually requires a sample size larger than 3 to declare significance at the *FDR*=0.05 level. For the rest of the methods, the highest number of significant AS events was found by Cufflinks, followed by MATS and DEXSeq. The most conservative method was SplicingCompass as shown in Table 3.

**Table 3:** **The number of shared differentially spliced genes detected by the selected methods for the HeatT1 data set**

	**DiffSplice**	**Cuffdiff**	**DEXSeq**	**MATS**	**rDiff-param**	**SplicingCompass**
**DiffSplice**	48	12	7	6	2	0
**Cuffdiff**		306	27	48	14	1
**DEXSeq**			155	27	37	3
**MATS**				241	16	0
**rDiff-param**					93	0
**SplicingCompass**						31

The table contains the number of significant differential spliced genes that reported by each methods (number on the diagonal) and numbers that are shared with another method.

**Figure 5:**
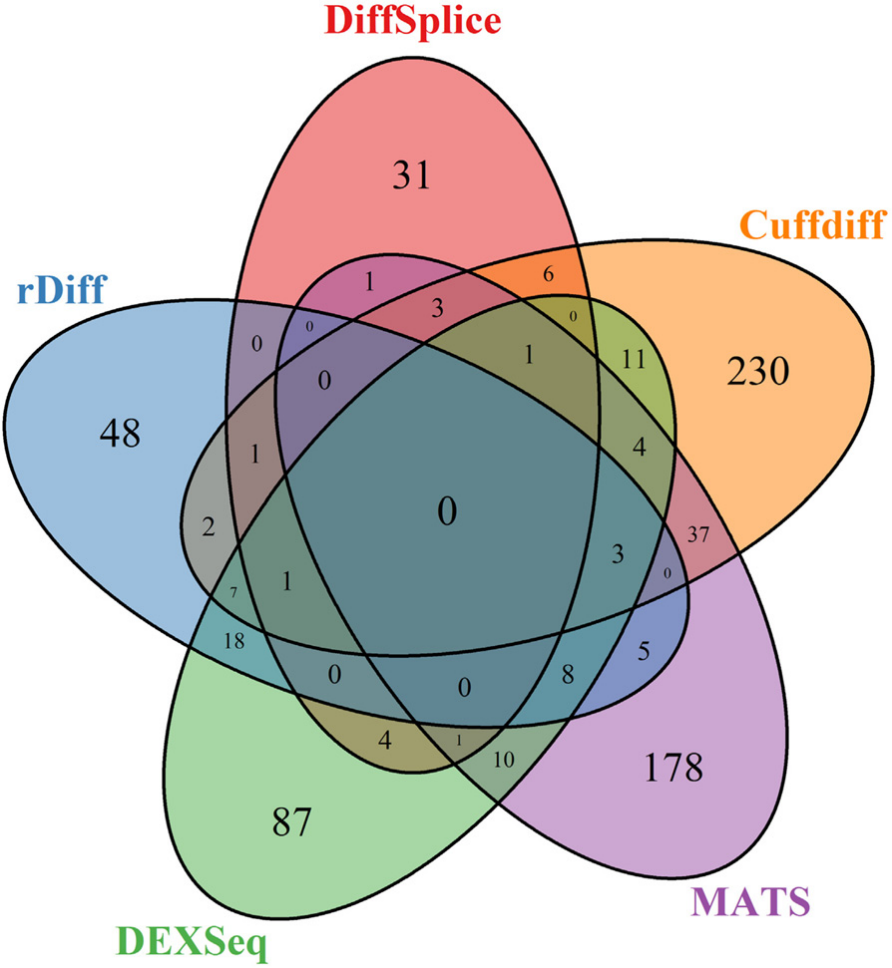
**Venn digram of heat shock data set.** Overlap among the set of DS genes found by 5 methods. SplicingCompass is not included because it almost shares nothing with other methods based on Table 3.

We also examined the overlaps of the set of significant AS genes found by each methods (Figure 5, Table 3). From Table 3, we noted that SplicingCompass was very conservative (having the smallest number of significant DS genes) and was also very “unique” in that it almost did not share any significant DS genes with other methods. The Venn diagram (Figure 5) did not include SplicingCompass. The results showed that the methods were very different from each other in that there was no gene that found by all five methods and that the proportion of genes that were found exclusively by each method was more than half. rDiff-parametric had 48.4% genes that were shared by at least one other methods. It was the only one that was close to 50% level. DEXSeq shared 40% of rDiff-parametric reported DS genes.

**Figure 6:**
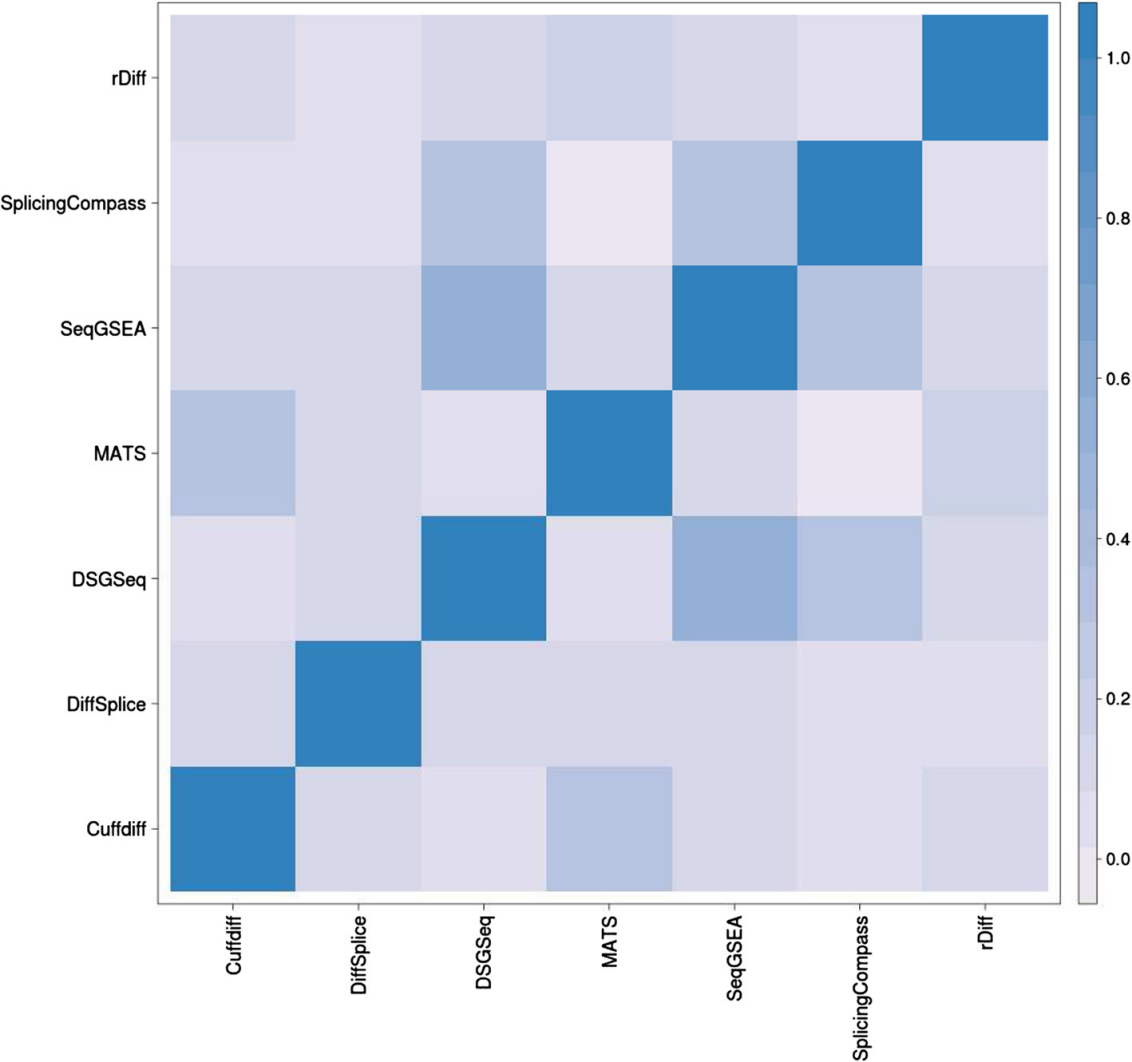
**Heat Map for correlation of the gene ranking scores obtained by the different methods for heat shock data set.** The correlations are generally low for any two methods, indicating the methods are very different. Two methods both using NB statistics (DSGseq and SeqGSEA) achieve the highest Spearman rank correlation of 0.52.

We further compared the results of all eight methods by investigating the correlation of gene ranking scores (computed as previously). We computed the Spearman rank correlations between all pairs of the eight methods and visualized it using a heat map (Figure 6). The correlations were calculated based on the ranking scores from 600 common genes that were reported by all methods. The highest correlation was observed between DSGseq and SeqGSEA as both methods use NB statistics (see Methods). Overall, the correlations were very low which indicated that these methods tended to rank genes differently with respect to alternative splicing.

### A list of experimentally validated AS genes which are known to exhibit AS in response to temperature changes

**Figure 7:**
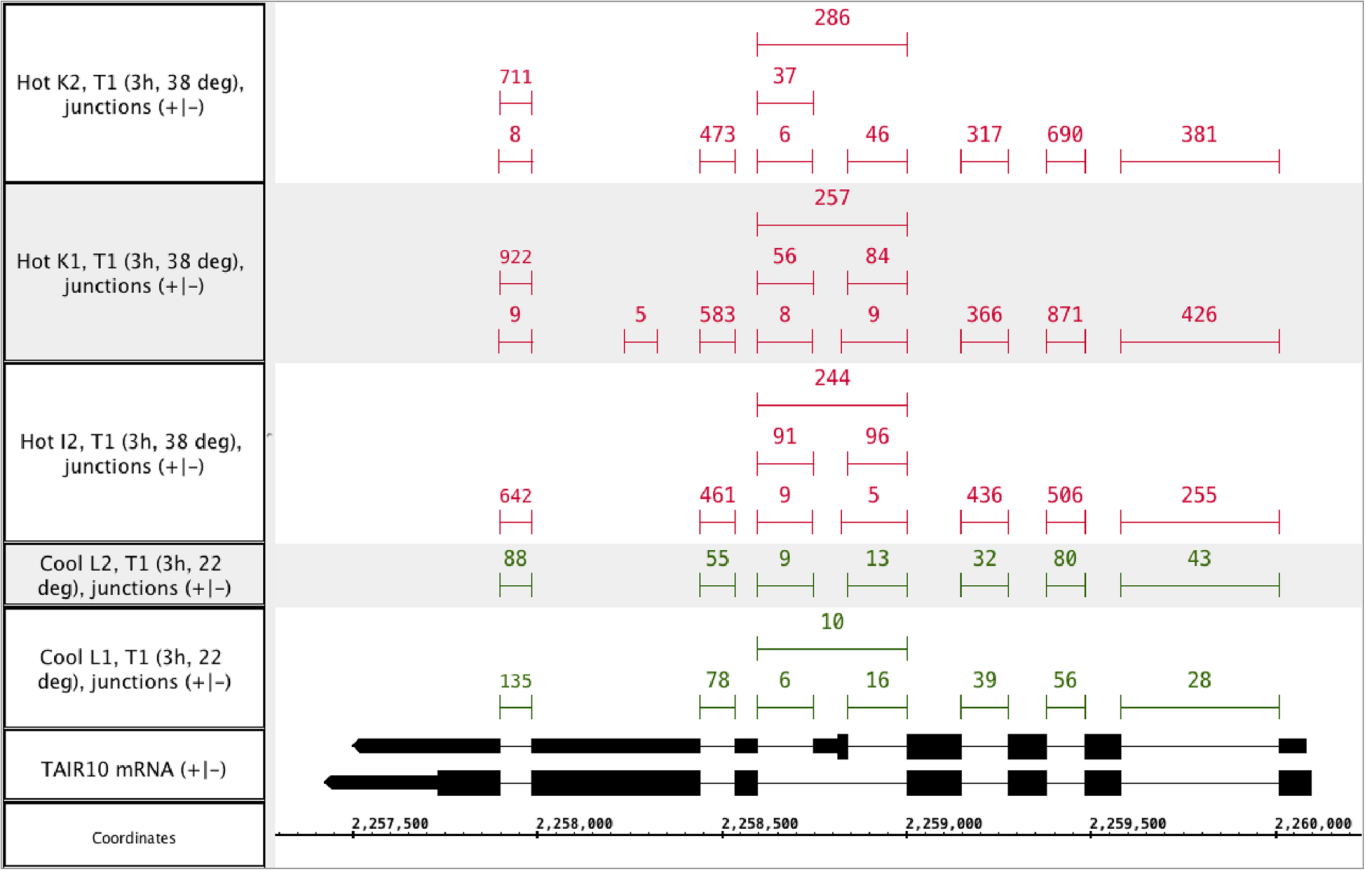
**SR45a.** Heat-induced differential splicing of Arabidopsis gene SR45a (AT1G07350) encoding an RNA-binding protein involved in splicing. Tracks labeled Hot and Cool contain exon-exon junction features inferred from spliced read alignments from heat-treated (hot) and control samples (cool). Junctions with fewer than five supporting reads are not shown. Two annotated gene models for SR45a are shown in the track labeled TAIR 10 mRNA. Taller blocks indicate translated regions of the gene model. Note that inclusion of an internal exon introduces a premature stop codon that interrupts translation and the exon-skipped form likely encodes the full-length protein. The gene is on the minus strand of chr1 and so transcription proceeds from right to left.

Since there have been studies that have linked some genes to alternatively spliced variants in response to heat stress, we came up with a list of six experimentally validated AS genes based on a search of the literature. AT1G01060 encodes LHY, a transcription factor involved in regulation of circadian rhythm. An A3SS event, encoding a 3-nt difference, has been found to occur as the ambient temperature changes [37]. This alternative splicing event has been confirmed by high resolution RT-PCR [37]. AT1G16610 encodes SR45, a member of SR proteins. AT1G16610 has two splice variants which differ by a 21-nt sequence which is present in SR45.1 but absent in SR45.2 [38]. It has been found that the relative abundance of SR45.2 is increased as temperature goes up [38]. Another two SR proteins, SR1/SR34 (AT1G02840) and SR30 (AT1G09140), have been reported to be alternatively spliced in response to heat stress [6,39-41]. In both cases, relevant transcripts differ by several hundred nts (337 nts in SR30 and 352 nts in SR1/SR34). All of the above AS events are A3SS. AT1G77080 encodes FLM, a MANS domain protein which regulates flowering. A mutually exclusive exon event has been found in this gene which is subject to temperature changes [42]. The P5CS1 gene (AT2G39800) contains an exon-3 skipping event that is subject to temperature variation [43]. The SR45a gene (AT1G07350) also contains an alternatively spliced internal exon and the proportion of exon-skipped transcript increases when exposure to heat stress. We illustrate the SR45a gene model and junction read alignments in different conditions using the Integrated Genome Browser [44] (Figure 7). Similar illustrations of the read pileups for the rest of genes are given in the Additional file 1.

At the cutoff *FDR*=0.05, MATS identified all seven genes and successfully located the actual genomic regions. DEXseq found two of them (SR1/SR34 and SR30) and Cufflinks reported one (FLM). None of the other methods were able to find these genes. For LHY and SR45, the A3SS events encompass a range of nt differences from a few to tens. MATS’s success in finding these events can probably be attributed to the exclusive use of junction reads. The small differences were easily overlooked by other methods that take into account of reads on full exonic regions. The junction reads that uniquely supported the A3SS events tend to be overwhelmed by the non-junction reads along the long exon (see the visualized read alignments in Additional file 1). DEXseq detect SR1/SR34 and SR30, with the differences in the A3SS events are several hundreds nt long. In DEXSeq, the junction reads are used as exon body reads.

### PCR validation of the real data set

In a separate study that will be described in detail (Loraine, A: Effects of heat and drought stresses on splicing and gene expression in Arabidopsis thaliana, submitted), we used semi-quantitative PCR to characterize heat induced splicing changes in seven genes that were annotated in TAIR 10 as being alternatively spliced. These seven genes thus provided a useful positive control for estimating the accuracy of the splicing analysis methods described here. These seven genes are AT1G77180, AT1G01490, AT2G02390, AT2G26670, AT3G19720, AT5G26780, AT1G09140. At the cutoff *FDR*=0.05, MATS reported five genes, followed by Cufflinks and DEXSeq, both of which picked out four genes. DSGseq, DiffSplice and rDiff identified one gene. The details about which methods picked out which genes and which AS events are contained in the seven genes are provided in Table 4.

**Table 4:** **The evaluation of the methods on the seven PCR validated genes**

**Gene**	**Found by which methods**	**AS events**
AT1G77180	DEXSeq, DSGseq, MATS	Alt acceptor in 5^′^ UTR
AT1G01490	None	Retained intron in 5^′^ UTR
AT2G02390	Cufflinks, DEXSeq, DiffSplice, MATS	4th exon alt acceptor
AT2G26670	Cufflinks, MATS	1st exon alt donor in coding region
AT3G19720	Cufflinks, MATS	Intron retention 3rd to last exon
AT5G26780	Cufflinks, DEXSeq	Intron retention last exon 3^′^ UTR
AT1G09140	DEXSeq, MATS, rDiff-param	Next to last exon alt acceptor

## Conclusions

In this paper, we have evaluated and compared eight methods for alternative/differential splicing analysis of RNA-seq data. The major observations for the AS methods are summarized in Table 5. These methods are classified into count-based models and isoform resolution models. Count-based models transform the question of AS analysis into the question of alternative usage of counting units while isoform resolution models seek to resolve the isoform relative abundances and in further compare the difference across conditions. Only Cufflinks and DiffSplice in our comparison belong to isoform resolution models. We’ve conducted both simulation studies and studies using real data to evaluate the methods. We created a customized simulation pipeline based on Flux Simulator. This pipeline allows users to repeat the simulation with different alternative splicing ratios, read depths and sample sizes.

**Table 5:** **Summary of the main observation for selected methods**

	**Class**	**Novel AS**	**Detection region**	**Comments**
**DiffSplice**	IR	Any type	ASM	Assembles transcriptome based on graph theory. Does not rely on annotation but does not use annotation either. The goodness of ASM is questionable. Generally low AUC. Performs poorly when detecting SE events.
**Cufflinks**	IR	Any type	Gene	Assembled transcripts merge with annotation to provide a more confident reference. Is least affected by incomplete annotation. Model is designed for pair-end data. Performs better for medium read depth than both low and high read depth. Performs better when detecting A3SS and A5SS events than other types of AS events. Computationally slow, but allows parallelization.
**DEXSeq**	CB	Only SE	Exon	Uses a generalized linear NB model. Achieves the highest AUC in many cases using accurate annotation. However, incomplete annotation can impose considerable problems for it. Poor FDR control.
**MATS**	CB	NS	AS event	Uses a Bayesian model. Solely based on junction reads. Can not detect complex AS events. Annotates splicing events with corresponding event types. Good FDR control in many simulation studies. Performs the best for real data.
**rDiff-param**	CB	NS	Gene	Conservative with default settings. Good FDR control but low AUC in many cases. Computationally fast.
**SplicingCompass**	CB	Only SE	Gene	Compares geometry angles of read count vectors. Generally poor FDR control and Medium AUC. Performs well when detecting SE events.
**DSGseq**	CB	Only SE	Gene	No p-value reported. Generally medium AUC. Performs well when detecting IR events and when using incomplete annotation. Computationally fast.
**SeqGSEA**	CB	Only SE	Gene	Integrates DE analysis with DS analysis. Generally high AUC. Requires a sample size around 5 to claim significance at a reasonable FDR level, i.e. *F* *D* *R*=0.05. Computation time increases dramatically as permutation times increases.

IR: Isoform resolution models. CB: Count based models. NS: Not Supported. ASM: Alternative Spliced Module.

From the perspective of AUC statistics, DEXSeq and DSGseq performed well in the simulation studies when the annotation is accurate and complete. DEXSeq was slightly better when two groups of samples were simulated using different dispersion parameters while DSGseq excelled when the same dispersion parameter is used. DSGseq is also more robust to changes in the AS ratio than DEXSeq. The drawback of DSGseq is that it does not calculate p-value. Both methods belongs to count based models. However, like other methods which depend on gene models, they performance was largely impaired when incomplete annotation was used. This may impose problems when working on non-model species or simply any species that are not well annotated. Cufflinks and DiffSplice are capable of assembling reads into transcripts and are thereby able to detect novel AS events. Only Cufflinks can take advantage of established gene models and is not fully dependent on the prior knowledge. These attributes render Cufflinks the best combination of accuracy and robustness against incomplete annotation. Therefore it is recommended for non-model species. On the other hand, Cufflinks achieves a better tradeoff between precision and recall. It also performs the best in an median read coverage of 60. The change of AS ratio affected methods’ discrimination power as well as the ability to control FDR. The rankings, however, were relatively stable as AS ratio changed, indicating that most methods is generally good enough to analyze real RNA-seq experiments where the splicing ratio might vary from gene to gene.

MATS uses a Bayesian framework to calculate the probability of a gene being alternatively spliced. Although MATS did not exhibit good performance under the evaluation of ROC curves and AUC, it was the best method under our comparison with respect to controlling the FDR at a proposed level. MATS excels in the precision of its results, which is very important for most biologists. The reason MATS had low recall and AUC is that MATS was only designed for detecting simple AS events. Therefore it was not satisfactory when the simulation included complex AS events. When only genes with simple AS events were involved, both recall and AUC improved dramatically for MATS. The superb performance of MATS in real data is boosted by the fact that all the 6 validated AS genes from the literature as well as for the 7 PCR validated AS genes are simple AS genes. rDiff-parametric also had a low FDR, however, but it appears to be due to its use of BF correction. In the analysis of heat shock RNA-seq data, MATS turned out to be the method that was the most consistent with the established experimental evidence as well as our PCR validations. The drawback of the MATS is that it is highly dependent on the goodness of annotation but it would be recommended for validating known AS events.

Large sample size (8 samples per condition) did not affect the discriminating power under ROC and/or AUC evaluation, but did improve several methods’ recall at the cost of decrease in precision. The several methods include Cufflinks, DEXSeq, SplicingCompass and especially SeqGSEA. SeqGSEA uses a permutation based approach to calculate p-values for genes being alternative spliced. It is likely that the p-values are scaled in accordance with sample size and we may expect a optimal sample size around 5 or 6 for using SeqGSEA. The sets of significantly alternatively spliced genes at given FDR threshold (FDR = 0.05) varied considerably between methods for the analysis of heat shock data. SeqGSEA and DSGseq had the highest correlations of the gene ranking scores due to using the same test statistics.

## Methods

### Parameter choices of software

All of the selected methods in this paper allow users to specify certain parameters. We have mostly used the default parameters as this is how most users apply these software packages. The detailed command lines and parameter choices used in the baseline simulation study are given in the Additional file 1. The version of each program used for the evaluations in the main paper is also given. For those that are implemented in R, including DEXSeq, SeqGSEA and SplicingCompass, it contains sample R code to run the analysis. For more detailed information, e.g., the meaning of the parameters and/or the whole list of parameters, we refer to the original publications.

For MATS, we used the mapping results instead of fastq files as the program input. Starting with MATS (3.0+), the program outputs two types of results: analysis based on both exon body reads as well as junctions reads and analysis based on junction reads alone. For all the comparisons, we used the latter but we showed in the Additional file 1 that there are only negligible differences in these two results.

For Cufflinks, we first assembled each sample individually using Cufflinks and then merged the resultant transcripts with annotation using Cuffmerge. The merged transcripts was used in Cuffdiff to perform the analysis of differential splicing. We used the fragment bias correction option in Cufflinks. In the analysis of heat shock data, the minimum number of replicates were set to 2 because one of the conditions has only two samples.

SeqGSEA integrates analysis regarding differential gene expression (DE) with analysis regarding differential splicing (DS). We only performed the latter and calculated the DS permutation p-values for 1000 iterations.

### Heat shock data sets

In the heat shock experiment [36], RNA was harvested from two experimental conditions (heat vs control) at two time points (T1 and T2). Previously grown in the same normal conditions, 3-week-old Arabidopsis plants were divided into 2 groups. In the heat shock group, plants were put into an incubator with temperature set to 38°C during a 3 h treatment. The first set of plants were collected immediately after the 3 h treatment and the second set of plants were harvested 24 h after the treatment. The first time point was designated as heat shock period and the second time point was designated as recovery period. In the control groups, the incubator was set to 22°C during the 3 h heat treatment and two sets of plants were collected from that incubator at T1 and T2 respectively. The RNA-Seq alignments used in this study are available for visualization in the Integrated Genome Browser via the IGB Quickload site http://www.igbquickload.org/abiotic. IGB is freely available from http://www.bioviz.org.

### Simulated RNA-seq data sets

We generated Arabidopsis RNAseq data using Flux Simulator [45] with exact ground truth expression levels. Arabidopsis is chosen because of its relatively small genome size and detailed genomic annotation. Two real data sets, Heat shock T1 and Heat shock T2, each with three replicates were used for generating simulated data. There was a good agreement between the simulated data by NB distributions and real data (Additional file 1: Figure S2).

We created a custom simulation pipeline (see Additional file 1) to create synthetic Arabidopsis RNA-seq data simulating different conditions. Flux Simulator is a single sample generator which carries out in-silico RNA-seq experiments. It starts with a random transcript population and then carries out library construction processes. Finally, it simulates the sequencing process including size selections, and platform-specific base calling errors. Our simulation pipeline extends the Flux Simulator capabilities to simulating differential splicing on two conditions with biological replicates. The simulation is a two-step workflow (Additional file 1: Figure S1). 1) First, we set empirical total transcript copy numbers for each gene and each sample based on real data and randomly choose genes for differential splicing across the conditions. The number of simulated replicates can be specified by the user. 2) Second, the transcript-level abundances are calculated based on the previous total transcript copy numbers, relative isoform proportions, and sequencing depth. Then, Flux Simulator can generate in-silico RNA-seq reads based on transcript-level abundances.

The custom simulation pipeline generated 100bp paired-end reads in fastq format. The relatively long read length (100bp) was deliberately chosen to produce more reads that cross exon-exon junctions. The generated synthetic reads were then mapped against the latest Arabidopsis genome TAIR 10 using the GMAP and GSNAP packages (version 2013-05-09) [46]. To maximize GSNAP’s ability to find spliced alignments, we used the RIKEN Arabidopsis full length cDNA sequences [47]. These sequences were utilized by GMAP with an option “-f”that looked for all possible splice sites and reported them to GSNAP as a database of known splice sites. The alignment results were output in SAM/BAM format which can be used for the subsequent alternative splicing analysis.

### Evaluation of the software results

We defined ranking scores for each method directly from the output. This score is a direct reflection of significance or evidence for alternative splicing across two conditions. For the six methods that provide adjusted p-values after multiple testing correction, we defined the score as 1−*P*_*adj*_. Rdiff use Bonferroni correction while SplicingCompass, MATS, DEXSeq, SeqGSEA and Cufflinks-Cuffdiff use Benjamini-Hochberg correction. DiffSplice and DSGseq do not provide p-values, and so we used their test statistics as the ranking scores: square root of *JSD* for DiffSplice and NB statistics for DSGseq (see the Methods overview in Background).

## Additional files

Additional file 1 **Supplementary material.** Contains supplementary figures referred to in the text. Here we also illustrate the simulation pipeline, and we compare the distribution and dispersion between simulated data and real data. The file also contains sample command lines or R code and computational time requirements for running each program. Finally the direct read alignments for the seven experimentally validated AS genes are shown in this file.

Additional file 2 **Results of eight selected programs on real data.** TXT file contains the result of each program run on the heat stress data set.

## References

[1] Black DL (2003). **Mechanisms of alternative pre-messenger RNA splicing**. Annu Rev Biochem.

[2] Lareau LF, Green RE, Bhatnagar RS, Brenner SE (2004). **The evolving roles of alternative splicing**. Curr Opin Struct Biol.

[3] Syed NH, Kalyna M, Marquez Y, Barta A, Brown JW (2012). **Alternative splicing in plants–coming of age**. Trends Plant Sci.

[4] Keren H, Lev-Maor G, Ast G (2010). **Alternative splicing and evolution: diversification, exon definition and function**. Nat Rev Genet.

[5] Graveley BR, Brooks AN, Carlson JW, Duff MO, Landolin JM, Yang L, Artieri CG, van Baren MJ, Boley N, Booth BW, Brown JB, Cherbas L, Davis CA, Dobin A, Li R, Lin W, Malone JH, Mattiuzzo NR, Miller D, Sturgill D, Tuch BB, Zaleski C, Zhang D, Blanchette M, Dudoit S, Eads B, Green RE, Hammonds A, Jiang L, Kapranov P (2011). **The developmental transcriptome of Drosophila melanogaster**. Nature.

[6] Reddy AS (2007). **Alternative splicing of pre-messenger RNAs in plants in the genomic era**. Annu Rev Plant Biol.

[7] Reddy AS, Rogers MF, Richardson DN, Hamilton M, Ben-Hur A (2012). **Deciphering the plant splicing code: experimental and computational approaches for predicting alternative splicing and splicing regulatory elements**. Front Plant Sci.

[8] Marquez Y, Brown JW, Simpson C, Barta A, Kalyna M (2012). **Transcriptome survey reveals increased complexity of the alternative splicing landscape in Arabidopsis**. Genome Res.

[9] Lamesch P, Berardini TZ, Li D, Swarbreck D, Wilks C, Sasidharan R, Muller R, Dreher K, Alexander DL, Garcia-Hernandez M, Karthikeyan AS, Lee CH, Nelson WD, Ploetz L, Singh S, Wensel A, Huala E (2012). **The Arabidopsis Information Resource (TAIR): improved gene annotation and new tools**. Nucleic Acids Res.

[10] Richardson DN, Rogers MF, Labadorf A, Ben-Hur A, Guo H, Paterson AH, Reddy AS (2011). **Comparative analysis of serine/arginine-rich proteins across 27 eukaryotes: insights into sub-family classification and extent of alternative splicing**. PLoS ONE.

[11] Reddy AS, Day IS, Gohring J, Barta A (2012). **Localization and dynamics of nuclear speckles in plants**. Plant Physiol.

[12] Wang B-B, Brendel V (2006). **Genomewide comparative analysis of alternative splicing in plants**. Proc Natl Acad Sci.

[13] Campbell MA, Haas BJ, Hamilton JP, Mount SM, Buell CR (2006). **Comprehensive analysis of alternative splicing in rice and comparative analyses with Arabidopsis**. BMC Genomics.

[14] Xiao YL, Smith SR, Ishmael N, Redman JC, Kumar N, Monaghan EL, Ayele M, Haas BJ, Wu HC, Town CD (2005). **Analysis of the cDNAs of hypothetical genes on Arabidopsis chromosome 2 reveals numerous transcript variants**. Plant Physiol.

[15] Alamancos GP, Agirre E, Eyras E (2013). **Methods to study splicing from high-throughput RNA Sequencing data**. Methods Mol Biol.

[16] Chen L (2013). **Statistical and computational methods for high-throughput sequencing data analysis of alternative splicing**. Stat Biosci.

[17] Pachter L: **Models for transcript quantification from RNA-Seq**. *arXiv:1104.3889v2*2011. http://arxiv.org/abs/1104.3889.

[18] Steijger T, Abril JF, Engstrom PG, Kokocinski F, Abril JF, Akerman M, Alioto T, Ambrosini G, Antonarakis SE, Behr J, Bertone P, Abril JF, Akerman M, Alioto T, Ambrosini G, Antonarakis SE, Behr J, Bertone P, Bohnert R, Bucher P, Cloonan N, Derrien T, Djebali S, Du J, Dudoit S, Engstrom P, Gerstein M, Gingeras TR, Gonzalez D, Grimmond SM (2013). **Assessment of transcript reconstruction methods for RNA-seq**. Nat Methods.

[19] Hayer K, Pizzaro A, Lahens N, Hogenesch J, Grant G: **Benchmark analysis of algorithms for determining and quantifying full-length mRNA splice forms from RNA-seq data**. *BioRxiv*2014. http://dx.doi.org/10.1101/007088.. 10.1093/bioinformatics/btv488 PMC4673975 26338770

[20] Wu J, Akerman M, Sun S, McCombie WR, Krainer AR, Zhang MQ (2011). **SpliceTrap: a method to quantify alternative splicing under single cellular conditions**. Bioinformatics.

[21] Katz Y, Wang ET, Airoldi EM, Burge CB (2010). **Analysis and design of RNA sequencing experiments for identifying isoform regulation**. Nat Methods.

[22] LeGault LH, Dewey CN (2013). **Inference of alternative splicing from RNA-Seq data with probabilistic splice graphs**. Bioinformatics.

[23] Singh D, Orellana CF, Hu Y, Jones CD, Liu Y, Chiang DY, Liu J, Prins JF (2011). **FDM: a graph-based statistical method to detect differential transcription using RNA-seq data**. Bioinformatics.

[24] Brooks AN, Yang L, Duff MO, Hansen KD, Park JW, Dudoit S, Brenner SE, Graveley BR (2011). **Conservation of an RNA regulatory map between Drosophila and mammals**. Genome Res.

[25] Anders S, Reyes A, Huber W (2012). **Detecting differential usage of exons from RNA-seq data**. Genome Res.

[26] Wang W, Qin Z, Feng Z, Wang X, Zhang X (2013). **Identifying differentially spliced genes from two groups of RNA-seq samples**. Gene.

[27] Aschoff M, Hotz-Wagenblatt A, Glatting KH, Fischer M, Eils R, Kdonig R (2013). **SplicingCompass: differential splicing detection using RNA-seq data**. Bioinformatics.

[28] Shen S, Park JW, Huang J, Dittmar KA, Lu ZX, Zhou Q, Carstens RP, Xing Y (2012). **MATS: a Bayesian framework for flexible detection of differential alternative splicing from RNA-Seq data**. Nucleic Acids Res.

[29] Drewe P, Stegle O, Hartmann L, Kahles A, Bohnert R, Wachter A, Borgwardt K, Ratsch G (2013). **Accurate detection of differential RNA processing**. Nucleic Acids Res.

[30] Wang X, Cairns MJ (2014). **SeqGSEA: a bioconductor package for gene set enrichment analysis of RNA-seq data integrating differential expression and splicing.**. Bioinformatics.

[31] Trapnell C, Williams BA, Pertea G, Mortazavi A, Kwan G, van Baren MJ, Salzberg SL, Wold BJ, Pachter L (2010). **Transcript assembly and quantification by RNA-Seq reveals unannotated transcripts and isoform switching during cell differentiation**. Nat Biotechnol.

[32] Hu Y, Huang Y, Du Y, Orellana CF, Singh D, Johnson AR, Monroy A, Kuan PF, Hammond SM, Makowski L, Randell SH, Chiang DY, Hayes DN, Jones C, Liu Y, Prins JF, Liu J (2013). **DiffSplice: the genome-wide detection of differential splicing events with RNA-seq**. Nucleic Acids Res.

[33] Bullard JH, Purdom E, Hansen KD, Dudoit S (2010). **Evaluation of statistical methods for normalization and differential expression in mRNA-Seq experiments**. BMC Bioinformatics.

[34] Marioni JC, Mason CE, Mane SM, Stephens M, Gilad Y (2008). **RNA-seq: an assessment of technical reproducibility and comparison with gene expression arrays**. Genome Res.

[35] Jiang H, Wong WH (2009). **Statistical inferences for isoform expression in RNA-Seq**. Bioinformatics.

[36] Gulledge AA, Roberts AD, Vora H, Patel K, Loraine AE (2012). **Mining Arabidopsis thaliana RNA-seq data with Integrated Genome Browser reveals stress-induced alternative splicing of the putative splicing regulator SR45a**. Am J Bot.

[37] James AB, Syed NH, Bordage S, Marshall J, Nimmo GA, Jenkins GI, Herzyk P, Brown JW, Nimmo HG (2012). **Alternative splicing mediates responses of the Arabidopsis circadian clock to temperature changes**. Plant Cell.

[38] Zhang XN, Mount SM (2009). **Two alternatively spliced isoforms of the Arabidopsis SR45 protein have distinct roles during normal plant development**. Plant Physiol.

[39] Yan K, Liu P, Wu CA, Yang GD, Xu R, Guo QH, Huang JG, Zheng CC (2012). **Stress-induced alternative splicing provides a mechanism for the regulation of microRNA processing in Arabidopsis thaliana**. Mol Cell.

[40] Barta A, Kalyna M, Reddy AS (2010). **Implementing a rational and consistent nomenclature for serine/arginine-rich protein splicing factors (SR proteins) in plants**. Plant Cell.

[41] Tanabe N, Yoshimura K, Kimura A, Yabuta Y, Shigeokam S (2007). **Differential expression of alternatively spliced mRNAs of Arabidopsis SR protein homologs, atSR30 and atSR45a, in response to environmental stress**. Plant Cell Physiol.

[42] Pose D, Verhage L, Ott F, Yant L, Mathieu J, Angenent GC, Immink RG, Schmid M (2013). **Temperature-dependent regulation of flowering by antagonistic FLM variants**. Nature.

[43] Kesari R, Lasky JR, Villamor JG, Des Marais DL, Chen YJ, Liu TW, Lin W, Juenger TE, Verslues PE (2012). **Intron-mediated alternative splicing of Arabidopsis P5CS1 and its association with natural variation in proline and climate adaptation**. Proc Natl Acad Sci U S A.

[44] Nicol JW, Helt GA, Blanchard SG, Raja A, Loraine AE (2009). **The Integrated Genome Browser: free software for distribution and exploration of genome-scale datasets**. Bioinformatics.

[45] Griebel T, Zacher B, Ribeca P, Raineri E, Lacroix V, Guigo R, Sammeth M (2012). **Modelling and simulating generic RNA-Seq experiments with the flux simulator**. Nucleic Acids Res.

[46] Wu TD, Nacu S (2010). **Fast and SNP-tolerant detection of complex variants and splicing in short reads**. Bioinformatics.

[47] Seki M, Satou M, Sakurai T, Akiyama K, Iida K, Ishida J, Nakajima M, Enju A, Narusaka M, Fujita M, Oono Y, Kamei A, Yamaguchi-Shinozaki K, Shinozaki K (2004). **RIKEN Arabidopsis full-length (RAFL) cDNA and its applications for expression profiling under abiotic stress conditions**. J Exp Bot.

