# Supplementary material for: Comparisons of computational methods for differential alternative splicing detection using RNA-seq in plant systems
Source: BMC Bioinformatics. 2014 Dec 16;15(1):364. doi: 10.1186/s12859-014-0364-4 (PMC4271460; doi:10.1186/s12859-014-0364-4)
Supplement: Supplementary file 1 — Supplementary material. Contains supplementary figures referred to in the text. Here we also illustrate the simulation pipeline, and we compare the distribution and dispersion between simulated data and real data. The file also contains sample command lines or R code and computational time requirements for running each program. Finally the direct read alignments for the seven experimentally validated AS genes are shown in this file. [file 12859_2014_364_MOESM1_ESM.pdf]

Comparisons of computational methods for alternative splicing detection using RNA-seq in plant systems - Supplementary Material

Ruolin Liu, Ann E loraine and Julie A Dickerson

Contents

1 Simulation Pipeline **2**

1.1 Step 1: Simulating biological replicates 2

1.2 Step 2: Simulating differential splicing 2

2 Sanity check of synthetic data **5**

3 Command lines and parameter choices **7**

3.1 Cufflinks 7

3.2 DEXSeq 7

3.3 DiffSplice 8

3.4 DSGseq 8

3.5 MATS 9

3.6 SeqGSEA 9

3.7 SplicingCompass 9

4 Comparison of two different MATS results **11**

5 Computational time requirement **11**

6 Visualization of read alignments in heat shock data for experimentally validated AS genes **12**

6.1 LHY 12

6.2 SR45 12

6.3 SR1/SR34 13
6.4 SR30 14
6.5 P5CS1 15
6.6 FLM 16

7 Supplementary figures **17**

1 Simulation Pipeline

1.1 Step 1: Simulating biological replicates

In order to approximate the situation in real RNA-seq experiment, we required two groups of empirical RNA-seq samples representing control and treatment groups respectively. First, the pipeline selected a random subset of genes that had more than one transcript based on annotation and that were expressed (have non-zero read counts in every replicate) in both input groups as true AS genes. The total transcripts copy number on a simulated gene was proportional to the number of reads counted on the real gene. We also introduced biological variance to gene expression by using Negative Binomial(NB) distributions. NB distribution is widely used for modeling variance across biological replicates. For each gene g we calculated mean μ_g and variance σ_g^2 of gene-level read counts across replicates and then performed a Loess regression f on the set of points (μ_g, σ_g^2). Thus we can borrow information across genes and do not rely on having large enough number of replicates to estimate variance. In the simulation studies with the same dispersion pattern we forced the regression function f to be the same under two conditions. For the simulation studies using different dispersion patterns the regression function f was learned from each of the two input groups and thus it differed for the two simulated conditions. The advantage of using Loess function is that Loess fitting does not make the same assumption of global homoscedasticity as general linear regression. Finally, the transcript counts for gene g were generated by NB distribution parameterized by mean μ_g and fitted variance $f(\mu_g)$.

1.2 Step 2: Simulating differential splicing

We defined a parameter, $PALT$, to control the relative transcript abundances across conditions. $PALT$ stands for Percentage of ALTernative form, ranging from 0 to 1. The relative transcript abundances of a multi-isoform gene g which has i isoforms, denoted by $e_g = (e_g^1, ..., e_g^i)$, were decided through the following formulas.

- if g is a AS gene, then we set $e_g^j = PALT, if j = i$ and $e_g^j = \frac{1-PALT}{i-1}, if j \neq i$.
- if g is not a AS gene, then we draw the relative abundance from a standard uniform distribution $e_g^j \in uniform(0,1)$ with a constraint $\sum_{j=1}^{i} e_g^j = 1$

In addition, we introduced another parameter Read Depth(RD) to allow user to control the mean per-based read depth which is defined as: $L * N/T$ Where L is the read length; N is the number of reads mapped to transcriptome; T is the transcriptome size.

Therefore the final absolute transcript abundance in the custom transcriptome expression profile are the product of gene-level transcript counts from step 1, relative transcript abundances and read depth tuner which makes sure the desired read depth is generated. Finally, the program, Flux Simulator calls this profile to generate RNA-seq reads.

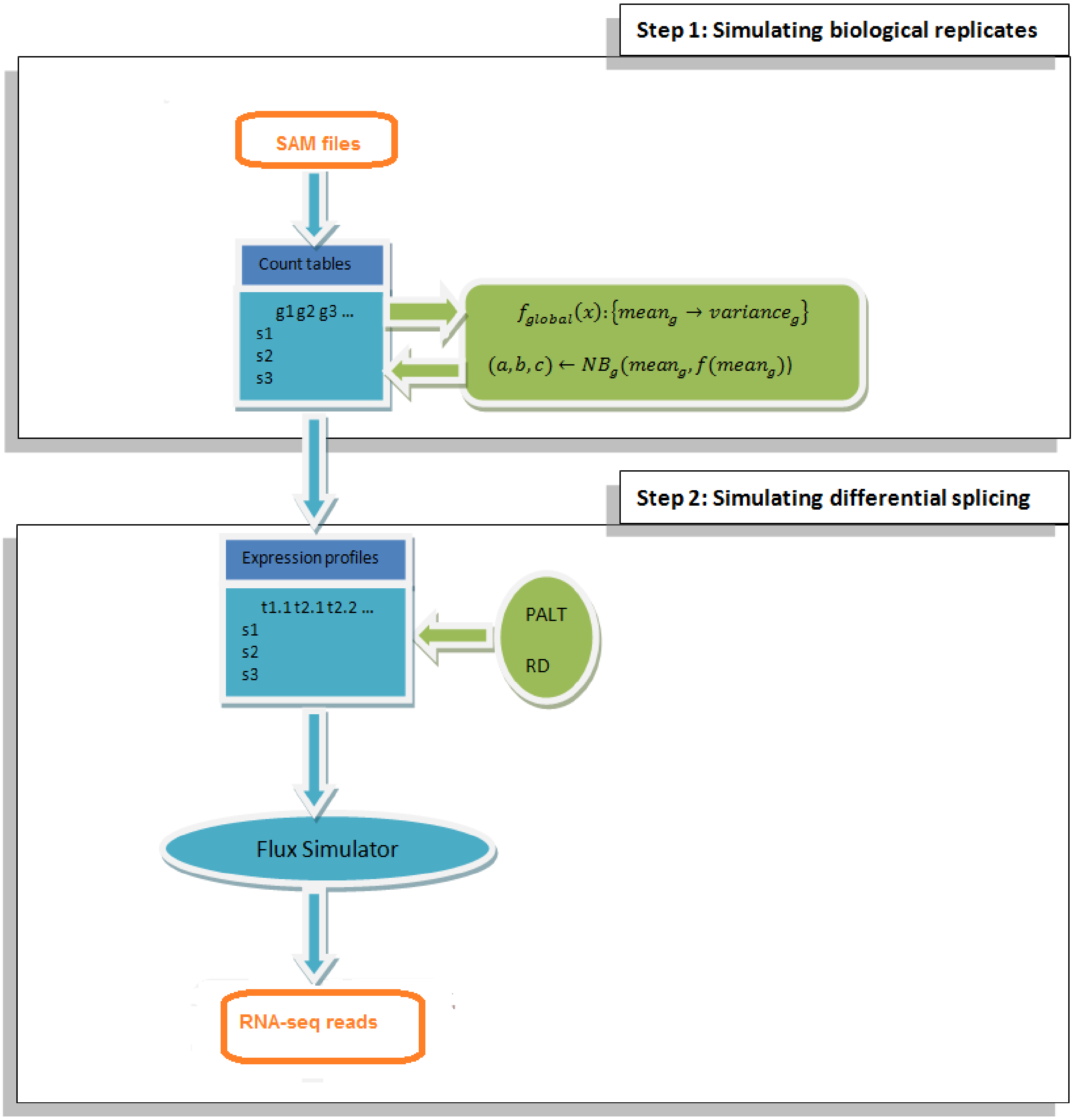

Figure S1: A two-step simulation pipeline. SAM files from real data are used as input for this pipeline. In the first step biological replicates are simulated by using Negative Binomial (NB) models. The raw fragment counts mean μ_g and variance σ_g^2 are calculated from the input. A regression function f is fitted on the set of points (μ_g, σ_g^2). Then the fitted variances are used as parameters in the NB models to generate three replicates, e.g. a, b, c. In the second step. The updated gene-level fragment counts are separate onto transcript levels based on the relative abundances and desired read depth. Finally, Flux Simulator is used to generated simulated RNA-seq reads.

2 Sanity check of synthetic data

To simulate biological replicates, we used Arabidopsis heat shock dataset [1] which contains three replicates for each of the two time points. The first time point was immediate after heat stress. The second was 24 h after recovery from the heat stress. The mean fragment counts across replicates and mean-variance relationship used in the simulation were estimated from the heat shock data set. Figure S2 shows the mean and variance of fragment counts in the log scale for synthetic data in baseline simulation study $RD100^H_D$ and heat shock data. There was a good agreement which indicated that the negative binomial model used in the simulation captured the mean-variance relationship or dispersion well. We further compared the distribution of the mean fragment counts in log scale. The simulation again captured the distribution in real data well.

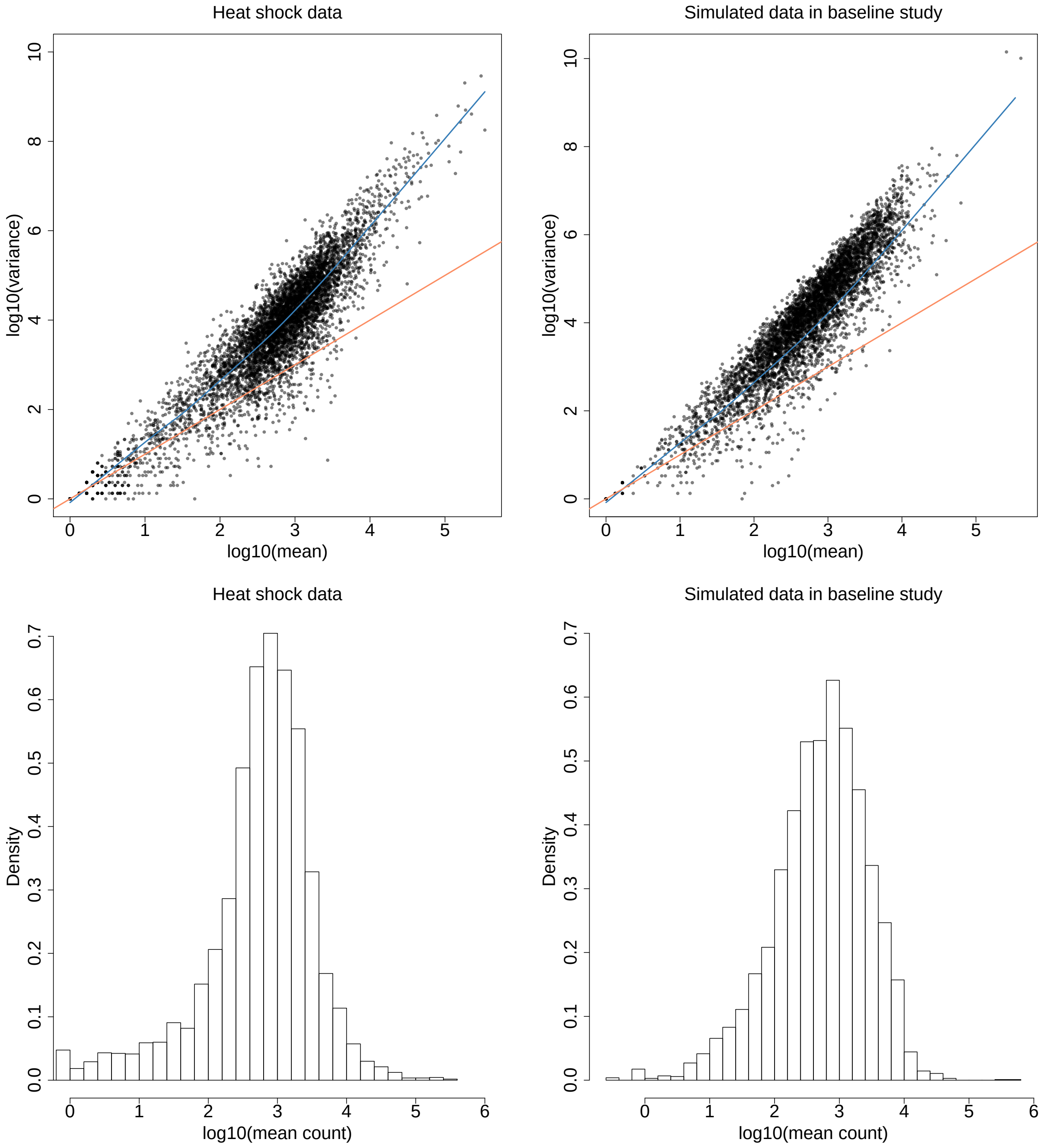

Figure S2: Comparison between real (left panels) and synthetic data (right panels). The 2 panels on top are scatter plots of mean-variance relationship across replicates. The blue lines are LOWESS regression lines. The orange lines are $variance = mean$ lines. It is clear that the real data is overdispersed with respect to what we would expect from a Poisson distribution and that it was well captured by a negative binomial distribution using in the simulated data. The two panels at the bottom compare the fragment counts distribution.

3 Command lines and parameter choices

3.1 Cufflinks

Cufflinks was written in Python and C++. It can be downloaded from http://cufflinks.cbcb.umd.edu/. We used the version 2.1.1 in this study. A newer version 2.2.0 was release while we were writing the paper.

```
cufflinks -p 8 -o RD100.control_r1 -L RD100C1 RD100.control_r1.sam
cufflinks -p 8 -o RD100.control_r2 -L RD100C2 RD100.control_r2.sam
cufflinks -p 8 -o RD100.control_r3 -L RD100C3 RD100.control_r3.sam
cufflinks -p 8 -o RD100.high.diff_r1 -L RD100HDM1 RD100.high.diff_r1.sam
cufflinks -p 8 -o RD100.high.diff_r2 -L RD100HDM2 RD100.high.diff_r2.sam
cufflinks -p 8 -o RD100.high.diff_r3 -L RD100HDM3 RD100.high.diff_r3.sam
cuffmerge -g TAIR10_GFF3_genes.gff -s TAIR10_nucleus.fas -p 8 assemblies.
    txt
cuffdiff -o diff_out -b TAIR10_nucleus.fas -L treatment,control -p 8 -
u merged_asm/merged.gtf RD100.high.diff_r1.sam,RD100.high.diff_r2.sam,
    RD100.high.diff_r3.sam RD100.control_r1.sam,RD100.control_r2.sam,RD100.
    control_r3.sam
```

3.2 DEXSeq

DEXSeq is a R package available in Bioconductor. We used the latest version 1.8.0 in this study.

```
library("DEXSeq")
inDir="countTables"
infile=c("RD100.high.diff_r1.count","RD100.high.diff_r2.count","RD100.high
    .diff_r3.count","RD100.control_r1.count","RD100.control_r2.count","
    RD100.control_r3.count")
setwd("countTables")
annotationfile=file.path("TAIR10_GFF3_genes_countingBin.gtf")
samples = data.frame(
condition = c(rep("treated", 3), rep("untreated", 3)),
replicate = c(1:3, 1:3),
row.names = c("g2_1","g2_2","g2_3","g1_1","g1_2","g1_3"),
stringsAsFactors = TRUE,
check.names = FALSE
)
samples$replicate=factor(samples$replicate)
ecs = read.HTSeqCounts(countfiles = file.path(inDir,infile),design =
    samples,flattenedfile = annotationfile)
ecs <- estimateSizeFactors(ecs)
ecs <- estimateDispersions(ecs)
ecs <- fitDispersionFunction(ecs)
ecs <- testForDEU(ecs)
res1 <- DEUresultTable(ecs)
sigExon=subset(res1, res1$padjust<0.05)
```

3.3 DiffSplice

DiffSplice was written in C++. It can be downloaded from http://www.netlab.uky.edu/p/bioinfo/DiffSplice/. We used the latest version 0.1.1 in this study.

```
diffsplice settings.cfg datafile.cfg output

## parameters used in settings.cfg
thresh_junction_filter_max_read_support     2
thresh_junction_filter_mean_read_support        0
thresh_junction_filter_num_samples_presence 0
ignore_minor_alternative_splicing_variants  yes
thresh_average_read_coverage_exon   0
thresh_average_read_coverage_intron 0
balanced_design_for_permutation_test        no
false_discovery_rate                0.05
thresh_foldchange_up                0.5
thresh_foldchange_down          0.5
thresh_sqrtJSD              0.1
```

3.4 DSGseq

DSGseq consists of a set of R scripts but is not a standard R packages. It can be downloaded from http://bioinfo.au.tsinghua.edu.cn/software/DSGseq/. We used the latest version 0.1.0.

```
bamToBed -i RD100.high.diff_r1.bam > RD100.high.diff_r1.bed
bamToBed -i RD100.high.diff_r2.bam > RD100.high.diff_r2.bed
bamToBed -i RD100.high.diff_r3.bam > RD100.high.diff_r3.bed
bamToBed -i RD100.control_r1.bam > RD100.control_r1.bed
bamToBed -i RD100.control_r2.bam > RD100.control_r2.bed
bamToBed -i RD100.control_r3.bam > RD100.control_r3.bed

SeqExpress count RD100.high.diff_r1.bed TAIR10.merge.refFlat RD100.high.
   diff_r1.count
SeqExpress count RD100.high.diff_r2.bed TAIR10.merge.refFlat RD100.high.
   diff_r2.count
SeqExpress count RD100.high.diff_r3.bed TAIR10.merge.refFlat RD100.high.
   diff_r3.count
SeqExpress count RD100.control_r1.bed TAIR10.merge.refFlat RD100.
   control_r1.count
SeqExpress count RD100.control_r2.bed TAIR10.merge.refFlat RD100.
   control_r2.count
SeqExpress count RD100.control_r3.bed TAIR10.merge.refFlat RD100.
   control_r3.count

Rscript DSGNB.R 3 RD100.high.diff_r1.count RD100.high.diff_r2.count RD100.
   high.diff_r3.count 3 RD100.control_r1.count RD100.control_r2.count
   RD100.control_r3.count RD100_high_diff.DSGresult
```

3.5 MATS

MATS was written Python. It can be downloaded from `http://rnaseq-mats.sourceforge.net/`. We used the latest version 3.0.8 in this study.

```
python RNASeq-MATS.py -b1 RD100.high.diff_r1.bam,RD100.high.diff_r2.bam,
   RD100.high.diff_r3.bam -b2 RD100.control_r1.bam,RD100.control_r2.bam,
   RD100.control_r3.bam -gtf TAIR10_GFF3_genes.gtf -t paired -len 100 -o
   MATS_OUT
```

3.6 SeqGSEA

SeqGSEA is a R package available in Bioconductor. We used the version 1.2.1. A newer version 1.5.0 was release while we were writing the paper.

```
library(SeqGSEA)
rm(list=ls())
case.pattern <- "^RD100.high"
ctrl.pattern <- "^RD100.control"
case.files <- dir("RD100.high.dm/seqgsea", pattern=case.pattern, full.
   names = TRUE)
control.files <- dir("RD100.control/seqgsea", pattern=ctrl.pattern, full.
   names = TRUE)
output.prefix <- "SeqGSEA.result"
library(doParallel)
cl <- makeCluster(2)
registerDoParallel(cl)
perm.times <- 1000
RCS <- loadExonCountData(case.files, control.files)
RCS <- exonTestability(RCS, cutoff=5)
geneTestable <- geneTestability(RCS)
RCS <- subsetByGenes(RCS, unique(geneID(RCS))[ geneTestable ])
geneIDs <- unique(geneID(RCS))
RCS <- estiExonNBstat(RCS)
RCS <- estiGeneNBstat(RCS)
permuteMat <- genpermuteMat(RCS, times=perm.times)
RCS <- DSpermutePval(RCS, permuteMat)
```

3.7 SplicingCompass

SplicingCompass is a R package. We used the latest version 1.0.1.

```
library("SplicingCompass")
packageDescription("SplicingCompass")
expInf=new("ExperimentInfo")
expInf=setDescription(expInf,"Group1 vs Group2")
expInf=setGroupInfo(expInf,
groupName1="ControlGroup1",sampleNumsGroup1=1:3,
```

```
groupName2="CaseGroup2",sampleNumsGroup2=4:6)
covBedCountFilesControl=c(
"RD100.control_r1.covBed",
"RD100.control_r2.covBed",
"RD100.control_r3.covBed")
covBedCountFilesCase=c(
"RD100.high.diff_r1.covBed",
"RD100.high.diff_r2.covBed",
"RD100.high.diff_r3.covBed")
junctionBedFilesControl=c(
"RD100.control_r1.juncBed",
"RD100.control_r2.juncBed",
"RD100.control_r3.juncBed")
junctionBedFilesCase=c(
"RD100.high.diff_r1.juncBed",
"RD100.high.diff_r2.juncBed",
"RD100.high.diff_r3.juncBed")
expInf=setCovBedCountFiles(expInf,c(covBedCountFilesCase,
   covBedCountFilesControl))
expInf=setJunctionBedFiles(expInf, c(junctionBedFilesCase,
   junctionBedFilesControl))
expInf=setReferenceAnnotation(expInf,"TAIR10_TableUnion.gtf")
referenceAnnotationFormat=list(IDFieldName="geneSymbol",idValSep=" ")
expInf=setReferenceAnnotationFormat(expInf,referenceAnnotationFormat)
checkExperimentInfo(expInf)
countTable=new("CountTable")
countTable=setExperimentInfo(countTable,expInf)
countTable=constructCountTable(countTable,printDotPerGene=TRUE)
sc = new("SplicingCompass")
sc = constructSplicingCompass(sc, countTable,
   minOverallJunctionReadSupport=3)
sc = initSigGenesFromResults(sc, adjusted=TRUE, threshold=0.05)
sigGenes = getSignificantGeneSymbols(sc)
resTab = getResultTable(sc)
```

rDIff-parametric

rDiff can be downloaded from `http://cbio.mskcc.org/public/raetschlab/user/drewe/rdiff/`. We used the latest version 0.3.

```
rdiff -o RD100HighDm -d data/ -a RD100.control_r1.bam,RD100.control_r2.bam
   ,RD100.control_r3.bam -b RD100.high.diff_r1.bam,RD100.high.diff_r2.bam,
   RD100.high.diff_r3.bam -g data/TAIR10_GFF3_genes.gff -m param -L 100
```

4 Comparison of two different MATS results

Table S1: MATS result using junction reads only versus result using both junction reads and exon body reads in simulation study $RD100_D^H$. The Pearson correlation of the p-values in these two results is as high as 0.978.

EventType	NumEvents.JC.only	SigEvents.JC.only	NumEvents.JC+ readsOnTarget	SigEvents.JC+ readsOnTarget
SE	704	153	704	152
MXE	14	1	14	1
A5SS	556	165	556	165
A3SS	1106	314	1106	313
RI	983	311	985	311

SE: Skipped exon
MXE: Mutually exclusive exon
A5SS: Alternative 5' splice site
A3SS: Alternative 3' splice site
RI: Retained intron
NumEvents.JC.only: total number of events detected using junction reads only
SigEvents.JC.only: number of significant events detected using junction reads only
NumEvents.JC+readsOnTarget: total number of events detected using both junction reads and exon body reads
SigEvents.JC+readsOnTarget: number of significant events detected using both junction reads and exon body reads

5 Computational time requirement

We ran the code shown in the previous section in Iowa State University super cluster called Lightning. The code was all executed in a single node and a single core with 16GB RAM. Although we used a cluster, this amount of computational power can be easily obtained in a standard PC. All the programs were finished within a few hours. The computation time required for SeqGSEA is largely affected by the permutation times. In this study, we set it to 1000. The total required CPU time for each method in the baseline simulation study $RD100_D^H$ is given in the Table S1.

Table S2: Total computational time in CPU-seconds

**Cufflinks**	**DEXSeq**	**MATS**	**SpComp**	**DSGseq**	**rDiff-param**	**DiffSplice**	**SeqGSEA**
41172s	6096s	8371s	10408s	1256s	1038s	4415s	39539s

6 Visualization of read alignments in heat shock data for experimentally validated AS genes

We have examined a few Arabidopsis genes that are known to be differentially spliced in response to ambient temperature changes. The following figures are the visualization of reads alignment of these few known genes using Integrated Genome Browser [4]. Solid bars represent reads, and thin lines indicate gaps in the alignment.

6.1 LHY

LATE ELONGATED HYPOCOTYL (LHY), circadian clock genes, are known to be differential spliced in response to temperature changes[2]. 5 transcripts have been found (based on TAIR10) in gene AT1G01060 which belongs to LHY gene family. Transcript AT1G01060.4 differs from other transcripts by 3-nt difference in the 3' site.

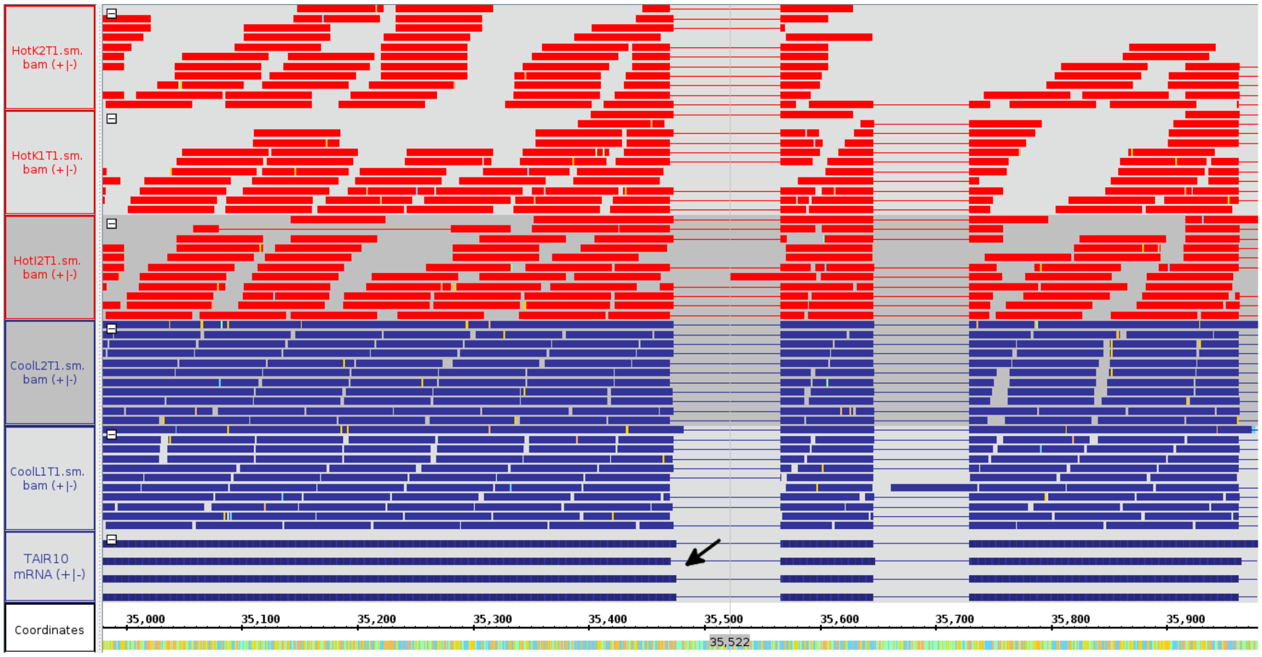

Figure S3: LHY. Visualization of read alignments in heat shock data. Reads from heat stress group are colored in red whereas reads from control groups are colored in blue. The black arrow indicates where the AS event happens in the gene model.

6.2 SR45

AT1G16610 encodes SR45 which is a member of SR protein family. A alternative 3'SS event differed by a 21-nt sequence has been found to occur as ambient temperature changes [7].

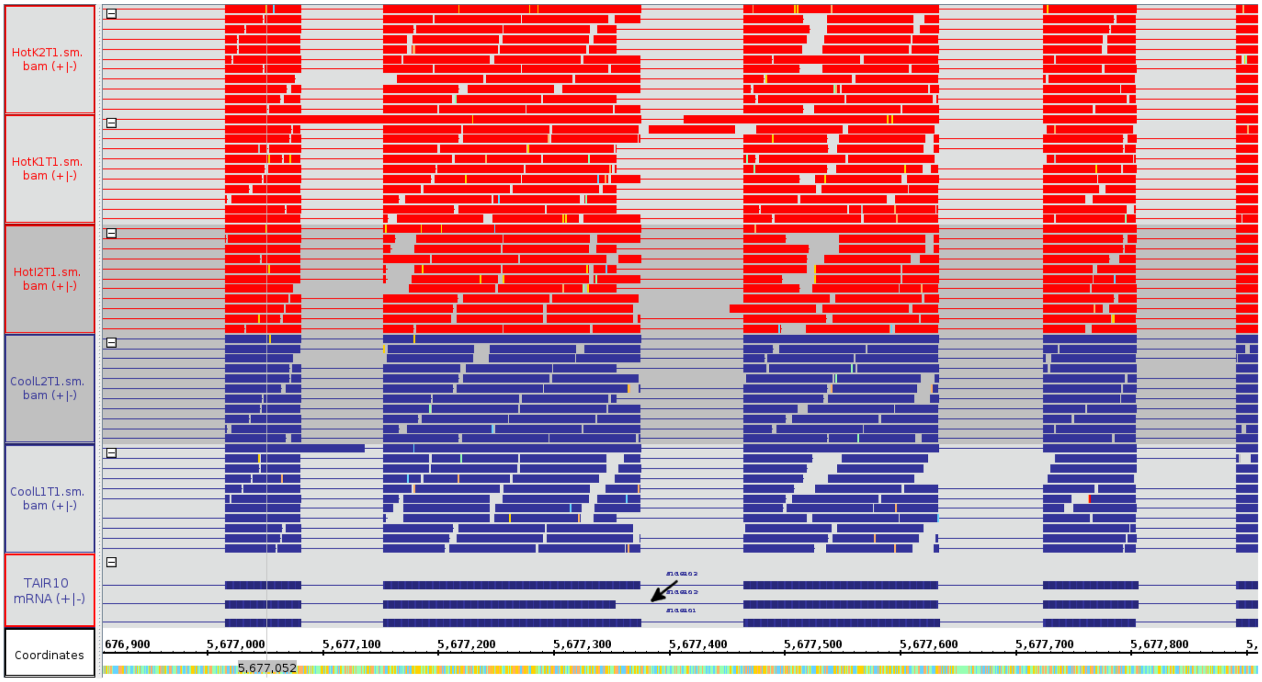

Figure S4: SR45. Visualization of read alignments in heat shock data. Reads from heat stress group are colored in red and reads from control groups are colored in blue. The black arrow indicates where the AS event happens in the gene model.

6.3 SR1/SR34

AT1G02840 encodes SR1/SR34 protein, a member of highly conserved family of spliceosome proteins. An alternative 3'SS event has been found as ambient temperature changes[6].

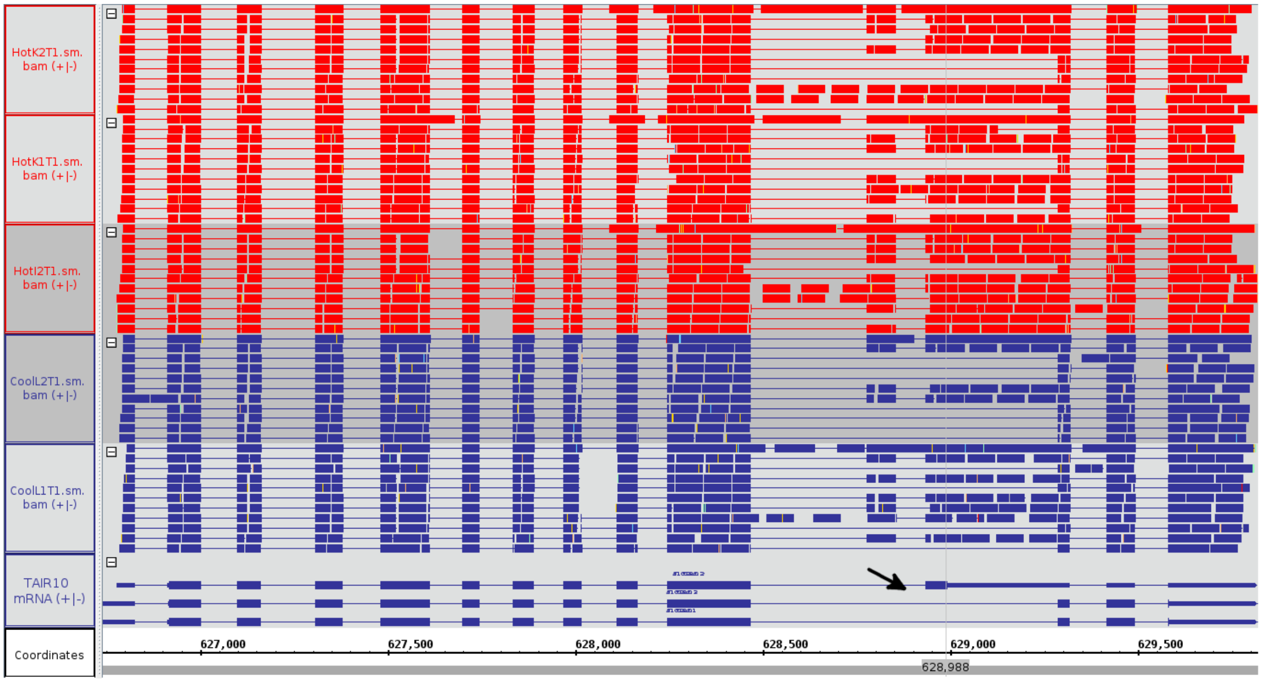

Figure S5: SR1/SR34. Visualization of read alignments in heat shock data. Reads from heat stress group are colored in red and reads from control groups are colored in blue. The black arrow indicates where the AS event happens in the gene model.

6.4 SR30

AT1G09140 encodes SR30, a member of highly conserved family of spliceosome proteins. An alternative 3'SS event has been found in response to heat stress [6].

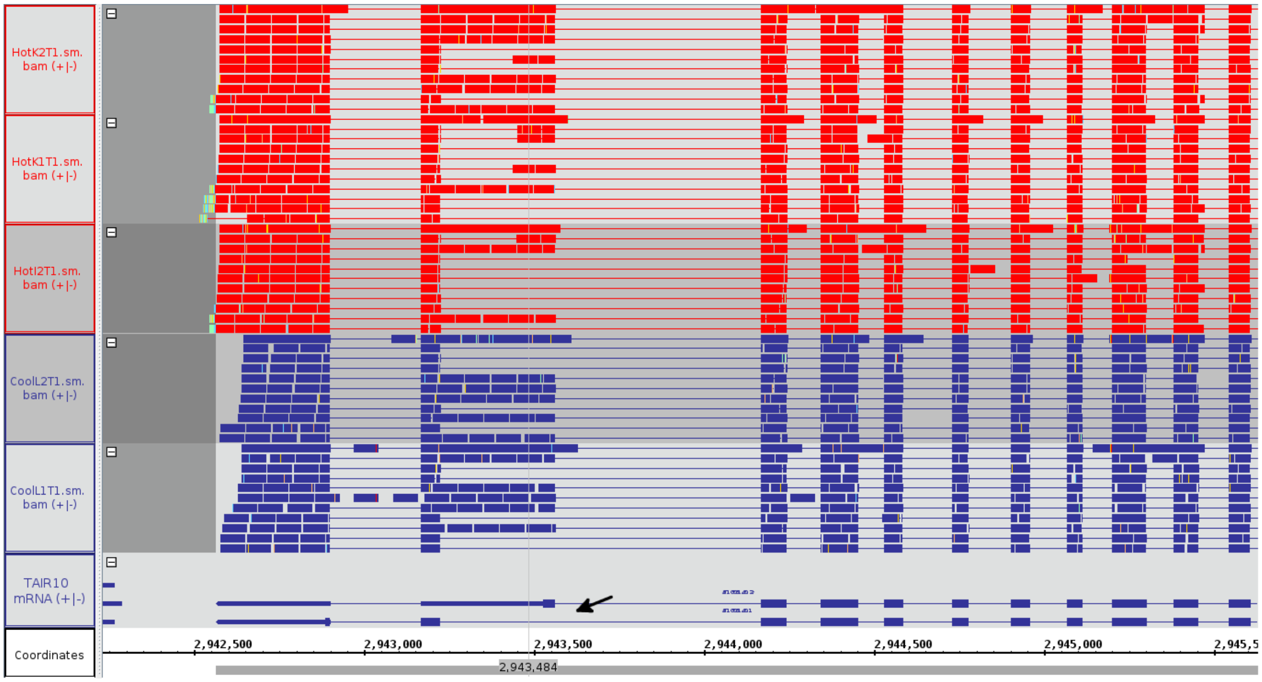

Figure S6: SR30. Visualization of read alignments in heat shock data. Reads from heat stress group are colored in red and reads from control groups are colored in blue. The black arrow indicates where the AS event happens in the gene model.

6.5 P5CS1

P5CS1 gene (AT2G39800) contains an exon-3 skipping event which is subject to temperature variation [3].

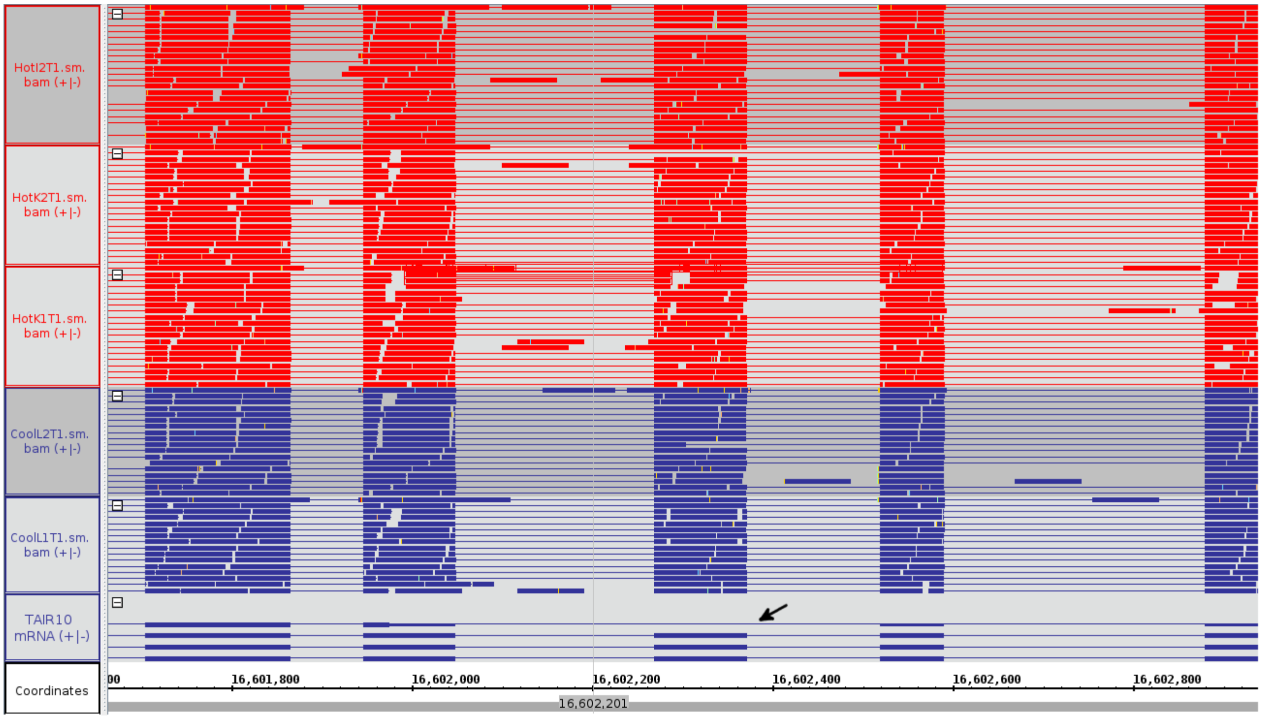

Figure S7: P5CS1. Visualization of read alignments in heat shock data. Reads from heat stress group are colored in red and reads from control groups are colored in blue. The black arrow indicates where the AS event happens in the gene model.

6.6 FLM

AT1G77080 encodes FLM, a protein which regulates flowering. An mutually exclusive exon event has been found in subject to temperature changes [5]

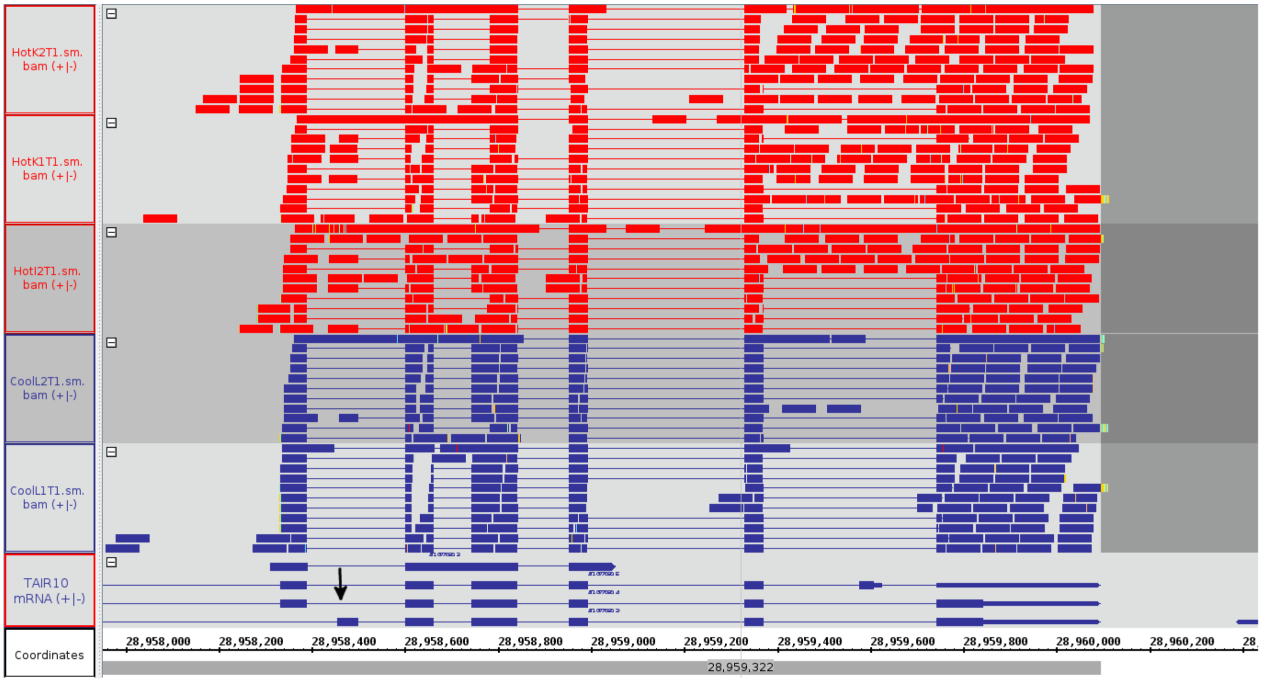

Figure S8: FLM. Visualization of read alignments in heat shock data. Reads from heat stress group are colored in red and reads from control groups are colored in blue. The black arrow indicates where the AS event happens in the gene model.

7 Supplementary figures

This section contains supplementary figures referred to in the main article.

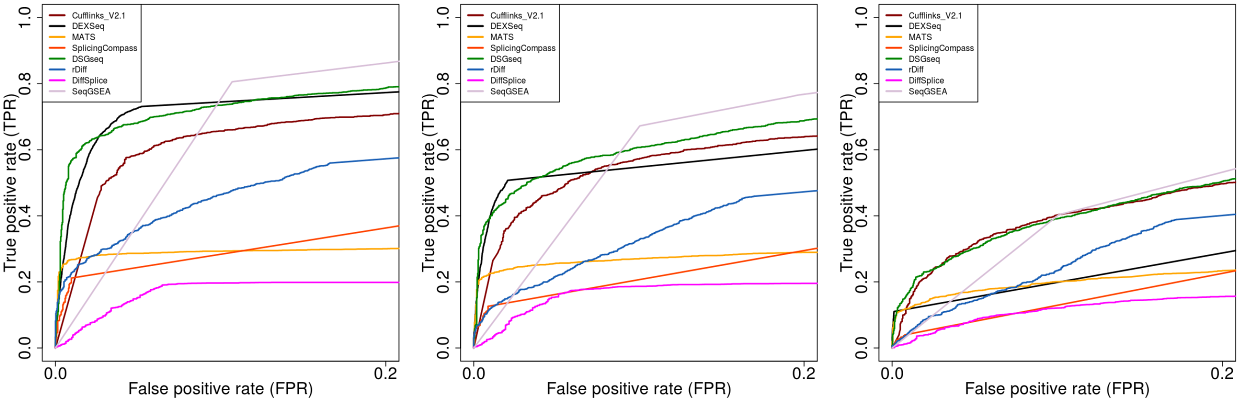

Figure S9: ROC curves evaluation for three different AS ratios when two groups of samples have the same dispersion pattern. ROC curves for simulation studies $\text{High}^{\text{Same}}_{\text{100x}}$ (left panel), $\text{Medium}^{\text{Same}}_{\text{100x}}$ (middle panel), $\text{Low}^{\text{Same}}_{\text{100x}}$(right panel). These ROC curves are obtained at a simple size of 3 for each condition.

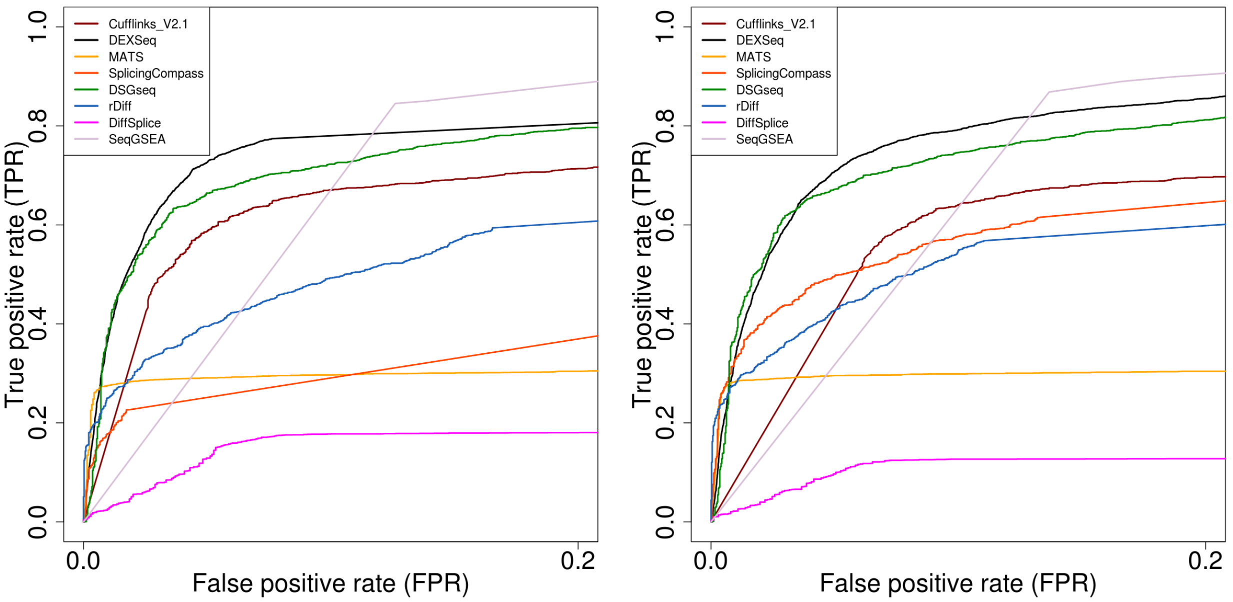

Figure S10: ROC curves evaluation for the two different samples sizes. Left panel shows ROC curves in the baseline simulation study $\text{High}^{\text{Diff}}_{100\text{x}} RD100^{H}_{D}$ which contained three replicates for each condition. The right panel shows the ROC curves when the sample size was increased to 8.

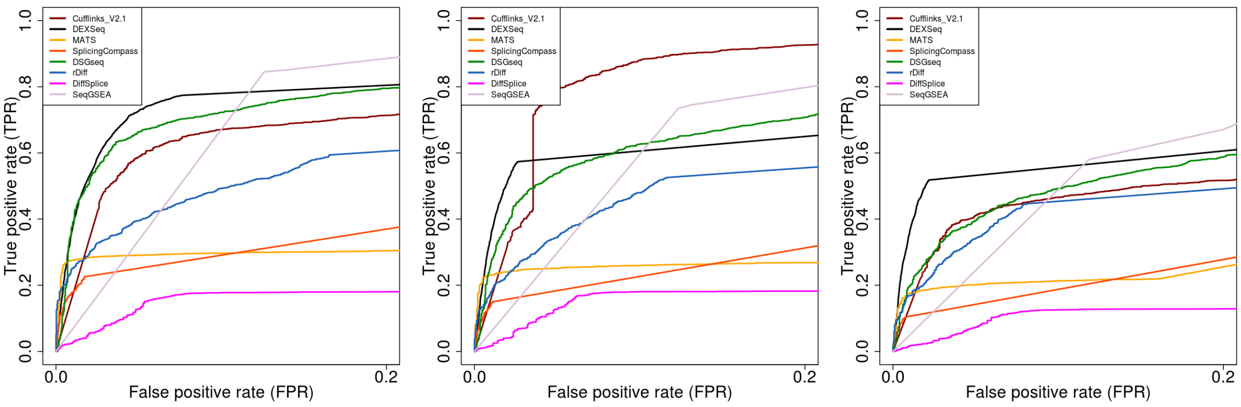

Figure S11: ROC curves evaluation for three different read depths, simulation studies $100\text{x}^{\text{Diff}}_{\text{High}}$ (left panel), $60\text{x}^{\text{Diff}}_{\text{High}}$ (middle panel), $25\text{x}^{\text{Diff}}_{\text{High}}$(right panel).

References

[1] A. A. Gulledge, A. D. Roberts, H. Vora, K. Patel, and A. E. Loraine. Mining Arabidopsis thaliana RNA-seq data with Integrated Genome Browser reveals stress-induced alternative splicing of the putative splicing regulator SR45a. *Am. J. Bot.*, 99(2):219–231, Feb 2012.

[2] A. B. James, N. H. Syed, S. Bordage, J. Marshall, G. A. Nimmo, G. I. Jenkins, P. Herzyk, J. W. Brown, and H. G. Nimmo. Alternative splicing mediates responses of the Arabidopsis circadian clock to temperature changes. *Plant Cell*, 24(3):961–981, Mar 2012.

[3] R. Kesari, J. R. Lasky, J. G. Villamor, D. L. Des Marais, Y. J. Chen, T. W. Liu, W. Lin, T. E. Juenger, and P. E. Verslues. Intron-mediated alternative splicing of Arabidopsis P5CS1 and its association with natural variation in proline and climate adaptation. *Proc. Natl. Acad. Sci. U.S.A.*, 109(23):9197–9202, Jun 2012.

[4] J. W. Nicol, G. A. Helt, S. G. Blanchard, A. Raja, and A. E. Loraine. The Integrated Genome Browser: free software for distribution and exploration of genome-scale datasets. *Bioinformatics*, 25(20):2730–2731, Oct 2009.

[5] D. Pose, L. Verhage, F. Ott, L. Yant, J. Mathieu, G. C. Angenent, R. G. Immink, and M. Schmid. Temperature-dependent regulation of flowering by antagonistic FLM variants. *Nature*, 503(7476):414–417, Nov 2013.

[6] K. Yan, P. Liu, C. A. Wu, G. D. Yang, R. Xu, Q. H. Guo, J. G. Huang, and C. C. Zheng. Stress-induced alternative splicing provides a mechanism for the regulation of microRNA processing in Arabidopsis thaliana. *Mol. Cell*, 48(4):521–531, Nov 2012.

[7] X. N. Zhang and S. M. Mount. Two alternatively spliced isoforms of the Arabidopsis SR45 protein have distinct roles during normal plant development. *Plant Physiol.*, 150(3):1450–1458, Jul 2009.
